# Supplementary material for: Protocol for a feasibility study of smoking cessation in the surgical pathway before major lung surgery: Project MURRAY
Source: BMJ Open. 2020 Nov 6;10(11):e036568. doi: 10.1136/bmjopen-2019-036568 (PMC7651715; doi:10.1136/bmjopen-2019-036568)
Supplement: Supplementary data [file bmjopen-2019-036568supp001.pdf]

APPENDIX

Appendix A – Major cardiopulmonary complications as classified by the European Society of Thoracic Surgeons (ESTS)

ARDS: Adult respiratory distress syndrome defined according to the American-European consensus conference. All of the following criteria should be met:
1. Acute onset
2. Arterial hypoxemia with PaO2/FIO2 ratio lower than 200 (regardless PEEP level)
3. Bilateral infiltrates at chest radiograph or CT scan
4. No clinical evidence of left atrial hypertension or pulmonary artery occlusive pressure <18 mmHg
5. Compatible risk factors

Atrial Arrhythmia: new onset of atrial fibrillation/flutter (AF) requiring medical treatment or cardioversion. Does not include recurrence of AF which had been present preoperatively.

Ventricular Arrhythmia: sustained ventricular tachycardia or ventricular fibrillation that has been clinically documented and treated by ablation therapy, implantable cardioverter defibrillator, permanent pacemaker, pharmacologic treatment or cardioversion.

Bronchoscopy for atelectasis: postoperative atelectasis documented clinically or radiographically that needed bronchoscopy.

Pneumonia: defined according to the last CDC criteria. Two or more serial chest radiographs with at least **one** of the following:

- New or progressive and persistent infiltrate
- Consolidation
- Cavitation

AND at least **one** of the following:

- Fever (>38°C or >100.4°F) with no other recognized cause
- Leukopenia (<4000 WBC/mm3) or leukocytosis (>12,000 WBC/mm3)
- For adults >70 years old, altered mental status with no other recognized cause

AND at least **two** of the following:

- New onset of purulent sputum, or change in character of sputum, or increased respiratory secretions, or increased suctioning requirements
- New onset or worsening cough, or dyspnea, or tachypnea

- Rales or bronchial breath sounds Worsening gas exchange (e.g. O2 desaturations (e.g., PaO2/FiO2 < 240), increased oxygen requirements, or increased ventilator demand).

Pulmonary embolism: confirmed by V/Q scan, angiogram or CT scan.

DVT: deep venous thrombosis confirmed by Doppler study, contrast study or other study and that required treatment.

Myocardial infarct: evidenced by one of the following criteria:

1. Transmural infarction diagnosed by the appearance of a new Q wave in two or more contiguous leads on ECG.
2. Subendocardial infarction (non Q wave) evidenced by clinical, angiographic electrocardiographic signs.
3. Laboratory isoenzyme evidence of myocardial necrosis.

Renal failure: defined as the onset of new renal failure in the postoperative period according to one of the following criteria:

1. Increase of serum creatinine to greater than 2.0, and 2-fold the preoperative creatinine level.
2. A new requirement for dialysis postoperatively.

Neurological complication: occurrence of one of the following central neurologic postoperative events not present preoperatively:

1. A central neurologic deficit persisting postoperatively for more than 72 hours
2. A transient neurologic deficit (transient ischemic attack or reversible ischemic neurological deficit) with recovery within 72 hours
3. A new postoperative coma persisting at least 24 hours and caused by anoxic/ischemic and/or metabolic encephalopathy, thromboembolic event or cerebral bleed

Appendix B – Seeley Systematic Classification of Morbidity and Mortality After Thoracic Surgery (TM &M) Classification of Severity

Complication: Any deviation from the normal postoperative course.

**Minor**	
Grade I	Any complication without need for pharmacologic treatment or other intervention.
Grade II	Any complication that requires pharmacologic treatment or minor intervention only.
**Major**	
Grade III	Any complication that requires surgical, radiologic, endoscopic intervention, or multi-therapy.
Grade IIIa	Intervention does not require general anaesthesia.
Grade IIIb	Intervention requires general anaesthesia.
Grade IV	Any complication requiring intensive care unit management and life support.
Grade IVa	Single organ dysfunction.
Grade IVb	Multi-organ dysfunction.
**Mortality**	
Grade V	Any complication leading to the death of the patient.

Appendix C – StEP Core Outcome Measures in Perioperative and Anaesthetic Care (COMPAC) – Post-operative Pulmonary Complications

Post-operative Pulmonary Complications*

Composite of respiratory diagnoses that share common pathophysiological mechanisms including pulmonary collapse and airway contamination:
(i) atelectasis detected on computed tomography or chest radiograph,
(ii) pneumonia using US Centers for Disease Control criteria,
(iii) Acute Respiratory Distress Syndrome using Berlin consensus definition,
(iv) pulmonary aspiration (clear clinical history ***AND*** radiological evidence).

***Exclusions**

Other diagnoses that do not share a common biological mechanism are best evaluated separately and only when clearly relevant to the treatment under investigation:
(i) pulmonary embolism,
(ii) pleural effusion,
(iii) cardiogenic pulmonary oedema,
(iv) pneumothorax,
(v) bronchospasm.

ARDS - Berlin definition

Timing: within 1 week of a known clinical insult or new or worsening respiratory symptoms
AND Chest imaging: bilateral opacities not fully explained by effusions, lobar/lung collapse or nodules
AND Origin of oedema: respiratory failure not fully explained by cardiac failure or fluid overload (requires objective assessment, e.g. echocardiography, to exclude hydrostatic oedema),
AND Oxygenation:
Mild PaO_2:FiO_2 between 26.7 - 40.0 kPa (200-300 mm Hg) with PEEP or CPAP $\geq$ 5 cm H_2O;
Moderate PaO_2:FiO_2 between 13.3 - 26.6 kPa (100-200 mm Hg) with PEEP $\geq$ 5 cmH_2O;
Severe PaO_2:FiO_2 $\leq$ 13.3 kPa (100 mm Hg) with PEEP $\geq$ 5 cm H_2O.
<u>Mechanical ventilation:</u>
The need for need for tracheal re-intubation and mechanical ventilation after extubation, and within 30 days after surgery OR mechanical ventilation for more than 24 h after surgery. The inclusion of non-invasive ventilation may be considered on a study-by-study basis.

BMJ Publishing Group Limited (BMJ) disclaims all liability and responsibility arising from any reliance placed on this supplemental material which has been supplied by the author(s)

Post-operative Pneumonia

Two or more serial chest radiographs with at least one of the following (one radiograph is sufficient for patients with no underlying pulmonary or cardiac disease):
(i) New or progressive and persistent infiltrates,
(ii) consolidation
(iii) cavitation;

AND at least **one** of the following:
(a) fever (>38°C) with no other recognised cause,
(b) leucopaenia (white cell count <$4x10^{9}$/1) or leucocytosis (white cell count >$12x10^{9}$/l),
(c) for adults >70 years old, altered mental status with no other recognised cause;

AND at least **two** of the following:
(a) new onset of purulent sputum or change in character of sputum, or increased respiratory secretions, or increased suctioning requirements,
(b) new onset or worsening cough, or dyspnoea, or tachypnoea,
(c) rales or bronchial breath sounds,
(d) worsening gas exchange (hypoxaemia, increased oxygen requirement, increased ventilator demand).

Lugg ST, *et al. BMJ Open* 2020; 10:e036568. doi: 10.1136/bmjopen-2019-036568
