# Supplementary material for: Protocol for a feasibility study of smoking cessation in the surgical pathway before major lung surgery: Project MURRAY
Source: BMJ Open. 2020 Nov 6;10(11):e036568. doi: 10.1136/bmjopen-2019-036568 (PMC7651715; doi:10.1136/bmjopen-2019-036568)
Supplement: Supplementary data [file bmjopen-2019-036568supp003.pdf]

[Insert Hospital Logo]

UNIVERSITY OF BIRMINGHAM

Study Number__________Site Number_________

A trial to study the effectiveness of sMoking cessation in the sURgical pathway befoRe mAjor lung sugerY: Project MURRAY: Feasibility Study

Participant Consent Form: Version 2.0

Principal Investigator:

Please initial box

1. I confirm that I have read and understand the patient information sheet (Version__Date__/__/__) for the above study. I have had the opportunity to consider the information, ask questions and have had these answered satisfactorily. ☐
2. I understand that my participation is voluntary and that I am free to withdraw at anytime without giving any reason, without my medical care or legal rights being affected. I give permission for data collected up until the point that I withdraw to be used in the research analysis. ☐
3. I understand that relevant sections of my medical notes and data collected during the study may be looked at by individuals from the research team, from regulatory authorities or from the NHS Trust, where it is relevant to my taking part in this research. I give permission for these individuals to have access to my records. ☐
4. I agree to be contacted either by telephone or post by a researcher to complete the research questionnaires at 4-8 weeks after surgery. ☐
5. I agree for my GP to be informed of my participation in the study. ☐
6. Optional – please choose option A) or B)

   A) I agree to take part in the interventional part of the study (including hospital based Intensive smoking cessation support). ☐

   B) I do not wish to take part in the interventional part of the study but I do agree to take part in the observational part of the study (including standard smoking cessation support). ☐
7. Optional - I agree for my name, address and contact details to be shared with the University of Birmingham and University College London, to be interviewed with audio recording and transcription at 4-8 weeks after surgery to discuss my treatment and participation in the study. I give permission that my anonymous quotes may be used in the reporting of the study. YES / NO ☐
8. I agree for my data to be used for ethically approved future research in lung surgery. ☐

______________________ ____________ ________________

Name of Participant **Date** **Signature**

______________________ ____________ ________________

Name of Person taking consent Date Signature

When completed: 1 for participant; 1 for researcher site file; 1 (original) to be kept in medical notes.

Project MURRAY: Feasibility Study: Participant Consent Form V2.0 (13/09/19) IRAS ID: 246910
