# Supplementary material for: Protocol for a feasibility study of smoking cessation in the surgical pathway before major lung surgery: Project MURRAY
Source: BMJ Open. 2020 Nov 6;10(11):e036568. doi: 10.1136/bmjopen-2019-036568 (PMC7651715; doi:10.1136/bmjopen-2019-036568)
Supplement: Supplementary data [file bmjopen-2019-036568supp002.pdf]

Insert Hospital Logo]

UNIVERSITY OF BIRMINGHAM

Study Number__________Site Number________

A trial to study the effectiveness of sMoking cessation in the sURgical pathway befoRe mAjor lung sugerY Project MURRAY: Feasibility Study

Consent Form: Staff interview: Version 2.0

Principal Investigator:

Please initial box

1. I confirm that I have read and understand the participant information sheet Version ___ Date __/__/__ concerning my participation in an interview/focus group for the above study. I have had the opportunity to consider the information, ask questions and have had these answered satisfactorily. ☐

2. I understand that my participation is voluntary and that I am free to withdraw at anytime without giving any reason, without my medical care or legal rights being affected. I give permission for data collected up until the point that I withdraw to be used in the research analysis. ☐

3. I agree for my personal information including name, address and phone number to be shared with the University of Birmingham and University College London to be interviewed with audio recording and transcription. ☐

4. I give permission that my anonymous quotes may be used in the reporting of the study. ☐

5. I agree to be interviewed/participate in a focus group for the above study. ☐

____________________	____________________	____________________
**Name of Participant**	**Date**	**Signature**
____________________	____________________	____________________
Name of Person taking consent	Date	Signature

When completed: 1 for participant; 1 for researcher site file; 1 (original) to be kept in medical notes.

Lugg ST, *et al. BMJ Open* 2020; 10:e036568. doi: 10.1136/bmjopen-2019-036568
