# Supplementary material for: Protocol for a feasibility study of smoking cessation in the surgical pathway before major lung surgery: Project MURRAY
Source: BMJ Open. 2020 Nov 6;10(11):e036568. doi: 10.1136/bmjopen-2019-036568 (PMC7651715; doi:10.1136/bmjopen-2019-036568)
Supplement: Author's manuscript [file bmjopen-2019-036568.draft_revisions.pdf]

BMJ Open

BMJ Open is committed to open peer review. As part of this commitment we make the peer review history of every article we publish publicly available.

When an article is published we post the peer reviewers' comments and the authors' responses online. We also post the versions of the paper that were used during peer review. These are the versions that the peer review comments apply to.

The versions of the paper that follow are the versions that were submitted during the peer review process. They are not the versions of record or the final published versions. They should not be cited or distributed as the published version of this manuscript.

BMJ Open is an open access journal and the full, final, typeset and author-corrected version of record of the manuscript is available on our site with no access controls, subscription charges or pay-per-view fees (http://bmjopen.bmj.com).

If you have any questions on BMJ Open's open peer review process please email info.bmjopen@bmj.com

BMJ Open

Protocol for a feasibility study of smoking cessation in the surgical pathway before major lung surgery: Project MURRAY

Journal:	*BMJ Open*
Manuscript ID	bmjopen-2019-036568
Article Type:	Protocol
Date Submitted by the Author:	19-Dec-2019
Complete List of Authors:	Lugg, Sebastian; University of Birmingham, Institute of Inflammation and Ageing<br>Kerr, Amy; University Hospitals Birmingham NHS Foundation Trust<br>Kadiri, Salma; University Hospitals Birmingham NHS Foundation Trust<br>Budacan, Alina; University Hospitals Birmingham NHS Foundation Trust<br>Farley, Amanda; University of Birmingham, Insitute of Applied Health Research<br>Perski, Olga; University College London, Institute of Epidemiology & Health<br>West, Robert; University College London, Institute of Epidemiology & Health<br>Brown, Jamie; University College London, Institute of Epidemiology & Health<br>Thickett , David ; University of Birmingham, Institute of Inflammation and Ageing<br>Naidu, Babu; University of Birmingham, Institute of Inflammation and Ageing
Keywords:	Cardiothoracic surgery < SURGERY, Adult thoracic medicine < THORACIC MEDICINE, Adult anaesthesia < ANAESTHETICS

SCHOLARONE™
Manuscripts

I, the Submitting Author has the right to grant and does grant on behalf of all authors of the Work (as defined in the below author licence), an exclusive licence and/or a non-exclusive licence for contributions from authors who are: i) UK Crown employees; ii) where BMJ has agreed a CC-BY licence shall apply, and/or iii) in accordance with the terms applicable for US Federal Government officers or employees acting as part of their official duties; on a worldwide, perpetual, irrevocable, royalty-free basis to BMJ Publishing Group Ltd ("BMJ") its licensees and where the relevant Journal is co-owned by BMJ to the co-owners of the Journal, to publish the Work in this journal and any other BMJ products and to exploit all rights, as set out in our licence.

The Submitting Author accepts and understands that any supply made under these terms is made by BMJ to the Submitting Author unless you are acting as an employee on behalf of your employer or a postgraduate student of an affiliated institution which is paying any applicable article publishing charge ("APC") for Open Access articles. Where the Submitting Author wishes to make the Work available on an Open Access basis (and intends to pay the relevant APC), the terms of reuse of such Open Access shall be governed by a Creative Commons licence – details of these licences and which Creative Commons licence will apply to this Work are set out in our licence referred to above.

Other than as permitted in any relevant BMJ Author's Self Archiving Policies, I confirm this Work has not been accepted for publication elsewhere, is not being considered for publication elsewhere and does not duplicate material already published. I confirm all authors consent to publication of this Work and authorise the granting of this licence.

For peer review only

TITLE

Protocol for a feasibility study of smoking cessation in the surgical pathway before major lung surgery: Project MURRAY

Corresponding author:

Mr Babu Naidu
Institute of Inflammation and Ageing, College of Medical and Dental Sciences,
University of Birmingham, B15 2TT
b.naidu@bham.ac.uk
Tel: +44 (0)121 371 3228

Sebastian T Lugg,[1,2] Amy Kerr,[1] Salma Kadiri,[1] Alina Budacan,[1] Amanda Farley,[3] Olga Perski,[4] Robert West,[4] Jamie Brown,[4] David Thickett,[1] Babu Naidu,[1] on behalf of the Project MURRAY Investigators

1. Institute of Inflammation and Ageing, College of Medical and Dental Sciences, University of Birmingham, Birmingham, UK.
2. Department of Thoracic Surgery, Heartlands Hospital, University Hospitals Birmingham NHS Foundation Trust, Birmingham, UK.
3. Institute of Applied Health Research, University of Birmingham, UK.
4. Institute of Epidemiology and Health, University College London, UK.

Keywords
Thoracic surgery, smoking cessation intervention, perioperative medicine, postoperative pulmonary complication, clinical trial

Word count 3998

For peer review only

ABSTRACT

Introduction: Smoking prior to major thoracic surgery is the biggest risk factor for development of postoperative pulmonary complications, with 1 in 5 patients continuing to smoke before surgery. Current guidance is that all patients should stop smoking before elective surgery yet very few are offered specialist smoking cessation support. Patients would prefer support within the thoracic surgical pathway. No study has addressed the effectiveness of such an intervention in this setting on cessation. The overall aim is to determine in patients who undergo major elective thoracic surgery whether an intervention integrated (INT) into the surgical pathway improves smoking cessation rates compared with usual care (UC) of standard community/hospital based NHS smoking support. This pilot study will evaluate feasibility of a substantive trial.

Methods and analysis: Project MURRAY is a trial comparing the effectiveness of INT and UC on smoking cessation. INT is pharmacotherapy and a hybrid of behavioural support delivered by the trained healthcare practitioners (HCPs) in the thoracic surgical pathway and a complimentary web-based application. This pilot study will evaluate the feasibility of a substantive trial and study processes in 5 adult thoracic centres including the University Hospitals Birmingham NHS Foundation Trust. The primary objective is to establish the proportion of those eligible who agree to participate. Secondary objectives include evaluation of study processes. Analyses of feasibility and patient-reported outcomes will take the form of simple descriptive statistics and where appropriate, point estimates of effects sizes and associated 95% CIs.

Ethics and dissemination: The study has obtained ethical approval from NHS Research Ethics Committee (REC number 19/WM/0097). Dissemination plan includes: informing patients and HCPs; engaging multidisciplinary professionals to support a proposal of a definitive trial and submission for a full application dependent on the success of the study.

Trial Registration Number: NCT04190966; Pre-results

For peer review only - http://bmjopen.bmj.com/site/about/guidelines.xhtml

Strengths and limitations of the study

- Smoking is common in patients undergoing major thoracic surgery and can result in significant economic and healthcare burden. Little is known about whether integrated smoking cessation support in the surgical pathway can improve smoking cessation rates.
- This study will assess the role of a hybrid model of integrated smoking cessation including both behavioral support and pharmacotherapy delivered by trained healthcare practitioners as well as use of a web-based application.
- This feasibility study will assess patient recruitment to inform a definitive study.
- Results from this study will add to the limited evidence towards of effective smoking cessation strategies in major thoracic surgery.
- This feasibility study will not answer the overarching research question of efficacy but will directly inform a well-designed definitive study

For peer review only

For peer review only - http://bmjopen.bmj.com/site/about/guidelines.xhtml

INTRODUCTION

Poorer outcomes for smokers in thoracic surgery

25,000 patients undergo major thoracic surgery every year in the UK (1). 1 in 5 patients smoke before surgery, which increases the risk of developing postoperative pulmonary complications including pneumonia and lung collapse. Just under half of smokers develop these complications with associated increased in-hospital mortality (0.5% to 12%), intensive therapy unit admissions (1.5% to 26%), increased hospital stay (5 to 14 days) and poorer long-term outcomes (2-4). Furthermore, lung cancer surgery patients have an 86% increased risk for cancer recurrence and two-fold decrease in 5-year survival compared with patients who quit smoking at diagnosis (5).

The need for integrated hospital-based smoking cessation support

The current NICE guidance is that smokers undergoing ‘elective’ surgery should receive behavioural support and stop-smoking pharmacotherapy as early as possible in their outpatient or pre-operative assessments. This should be offered weekly, preferably face-to-face, for a minimum of 4 weeks after the quit date (6). The Cochrane review supporting this type of ‘intensive’ intervention (7) was based on two small trials in orthopaedics and general surgery (8, 9).

However in current practice most thoracic surgery patients do not receive any pre-operative smoking cessation support. Of the 120 patients attending a large UK thoracic regional unit only 40% of current smokers were offered support (10), similar to the proportion of all eligible pre-operative patients agreeing to participate in smoking cessation studies (11, 12). Only 1 in 3 smokers self-report abstinence at the time of lung cancer surgery (4), with biochemical verification indicating much lower quit rates (7).

Thus the current usual care (UC) of referral to stop-smoking services does not meet the standard set by NICE. This may be due to these services being designed to promote long-term quitting, which many smokers undergoing surgery may not be willing to commit to. Many patients also report that attending smoking cessation clinic appointments during their work-up for surgery is a significant barrier to stopping smoking and would prefer bespoke support during hospital visits (13).

For peer review only - http://bmjopen.bmj.com/site/about/guidelines.xhtml

For peer review only

In pre-operative smoking cessation studies, behavioural support is delivered either by research nurses or independent smoking cessation practitioners (12, 14). It is logical that HCPs in the surgical pathway could be trained to deliver the support due to the high prevalence of smoking within this patient group. This approach is advocated by both the Lung Cancer Nurse Forum and the Society of Cardiothoracic Surgery (UK) and evidence suggests that nurse delivered smoking cessation interventions are effective (18). Timely access is also crucial; NICE lung cancer guidelines recommend that surgery should not be delayed to give up smoking (15) and needs to be performed within 62 days of presentation and 31 days of diagnosis to avoid heavy financial penalties on individual Trusts (16). Therefore avoiding delays in receiving behavioural and pharmacological support is of paramount importance.

Poor quality of available smoking cessation applications

A combination of a web-based application (web app) with structured face-to-face behavioural support may aid successful quit attempts, whilst also supporting HCPs in delivering smoking cessation support throughout the surgical pathway. A recent review of over 100 smoking cessation apps showed that only 6 were deemed to be of high quality (17). There are no apps specifically designed for smoking cessation in patients undergoing major surgery and none designed to provide hybrid support alongside a trained smoking cessation practitioner.

Summary

Many patients who undergo thoracic surgery continue to smoke, and effective pre-operative smoking cessation interventions may improve outcomes. However, there are few studies exploring strategies for integrating smoking cessation into the patient pathway, which is a high priority area for research in these patients. We have developed a bespoke, tailored intense, integrated smoking cessation intervention to test in a 'real-life' clinical trial within the UK. The intervention involves pharmacotherapy and a hybrid of support delivered by trained HCPs in the thoracic surgical pathway and a complementary web app. Support may be particularly effective because of site of the surgery, the lungs, makes this a clear 'teachable moment' for patients (18, 19).

For peer review only - http://bmjopen.bmj.com/site/about/guidelines.xhtml

Study aim

The overall aim of this research is to determine if integrated (INT) smoking cessation support in the surgical pathway improves smoking cessation rates in patients undergoing major elective thoracic surgery when compared with usual care (UC) of standard community/hospital based NHS smoking cessation support. To answer this research question with substantial evidence of the clinical and cost-effectiveness of INT approach, a multi-centre RCT is required. Feasibility studies are a recommended pre-requisite to assess feasibility of a large and expensive full-scale trial. We have therefore designed this multicentre feasibility study, which aims evaluate the feasibility of the INT by making the following quantitative and qualitative assessments.

Objectives of the feasibility study

The aims of the feasibility study are to assess various aspects of the trial design and management and not to determine the relative effectiveness of INT versus UC.

Primary Objective

To establish the proportion of those eligible who agree to participate in the study and receive the intervention.

Secondary Objectives

1. Assess the integration of the intervention and trial procedures into the clinical pathway by benchmarking metrics such as time from decision to operate to study recruitment and to cessation, in addition to and period of cessation pre- and post-operatively and relationship with treatment target dates.
2. Explore barriers to study recruitment, including reasons for non-participation from screening logs and infrastructure issues from 'benchmarking metrics'.
3. To pilot and fine-tune study procedures and data capture forms.
4. To describe the proportion of patients in the INT group who have quit smoking by the day of and/or one month after surgery.
5. To describe the proportion of patients in the 'observation only' UC group who have quit smoking by the day of surgery and/or one month after surgery.

For peer review only - http://bmjopen.bmj.com/site/about/guidelines.xhtml

6. To define the spread and variability of smoking cessation practices in patients in the INT group and in the UC group including pharmacotherapy use as reported by the patient.
7. To understand patients' experiences of and engagement with the intervention, and any unintended consequences.
8. To establish whether the intervention is acceptable to thoracic surgery patients and staff and investigate recommendations for optimisation of intervention delivery.

TRIAL DESIGN

Design

Project Murray is an RCT comparing the effectiveness of INT verses UC in smoking cessation rates in patients undergoing major thoracic surgery, taking form of a stepped wedge cluster randomised controlled trial (20), and as such will not require individual patient randomisation. This feasibility study will evaluate the substantive trial and study processes.

Setting

Trial recruitment will be over a period of 12 months with an additional 6-month follow-up period. Recruitment will initiate at the University Hospitals Birmingham NHS Foundation Trust, which is the trial co-ordinating site and performs >1000 major thoracic surgical procedures a year. Additional recruitment will involve 4 further regional thoracic surgical centres performing >400 major thoracic surgical procedures a year.

Flow of participants during the trial

The anticipated pathway of patients through the trial is shown in the trial schema (Figure 1). All adult patients who fulfil the inclusion and exclusion criteria during the study period will be approached and participant information sheets (PIS) will be provided. Written informed consent will be obtained after an opportunity for patients to discuss requirements for the study. If the patient accepts the intervention, they will be placed into the INT group of the study. If they decline the intervention they will be invited to take part in the observational only part of the study and will receive usual care (UC). We will extract routinely collected pre- and post-operative data from patient's medical records and also collect data using questionnaires. Adverse events will be collected throughout the duration of the study. The summary of assessments is detailed in (Table 1).

For peer review only - http://bmjopen.bmj.com/site/about/guidelines.xhtml

Study Eligibility

Inclusion Criteria

- Current tobacco smoker (smoked within the last 28 days)
- Major Thoracic Surgery (including both open and minimally invasive approach)
- Able to provide written informed consent
- At least 1 weeks' time to surgery
- Age over 18 years

Exclusion criteria

- Emergency thoracic surgery
- Inability to perform exhaled carbon monoxide (CO) measurements

Patient Identification and screening procedure

Patients who are listed for major thoracic surgery will be identified and screened for eligibility prior to surgery. If a patient is screened and not eligible for the study or does not consent to be in the intervention or observation group, a record of the case will be kept in the screening log and will inform recruitment targets. No further information will be collected on ineligible patients or those that have not given consent for participation in the study.

Patient Recruitment

The PIS, developed with feedback from our Patient and Public Involvement (PPI) representatives, will be sent to the patient before the initial consultation, allowing time for the patient to review and ask questions. As part of the normal consultation process, the HCP will deliver a brief intervention detailing the importance of smoking cessation as per standard clinical practise (National Centre for Smoking Cessation Training [NCSCT] - Ask, Advice, Act). Patients will then be asked to consider and consent to a trial testing the new bespoke integrated smoking cessation support delivered in secondary care by the surgical/nursing team. If a patient does not wish to enter the INT arm they will be approached to take part in the UC arm of the study.

For peer review only - http://bmjopen.bmj.com/site/about/guidelines.xhtml

If patients want to join the trial on the day of the clinic appointment, they can do so, as the intervention is low risk and aimed at being offered 'there and then'. A research team member will obtain written informed consent with delegated authority from the Principal Investigator. A copy of the signed consent form will be given to the participants and a copy will be placed in the medical notes. The original consent form will be stored in the investigators site file. Consent will be sought at every study contact and participants will be made aware that they are free to withdraw consent at any time without reprisal. Participants will be consented to inform their GP of their involvement in the study.

Intervention development

A smoking cessation package has been developed using best practice from NICE/NCSCT involving pharmacotherapy and a hybrid of behavioural support delivered by trained HCPs in the thoracic surgical pathway and a complementary web app. *'Quit4Surgery'* is a web app created using a user centred design approach and developed through a series of design workshops with patients, HCPs, academic researchers with expertise in smoking cessation and informed by behavioural frameworks/motivation theory. The iterative development was handled with 'Agile' and SCRUM project management (21, 22). *'Quit4Surgery'* contains behaviour change techniques that research suggests can improve the chances of quitting, including goal setting, self-monitoring, feedback, rewards, information about health consequences, advice on medication use, advice on changing routines, advice on coping and support for identity change (23). *'Quit4Surgery'* also enables HCPs to record and enter patients' CO levels.

Integration of full package of support into the surgical pathway (INT)

1) INT will be delivered by key HCPs in the surgical pathway (i.e. surgical nurses, lung cancer nurses, or pre-operative assessment nurses) who have received the NCSCT training to deliver behavioural support required for stop smoking practitioners with specific focus on major surgical patients (24). The initial consultation of 15-30 minutes will occur either on the day of consent or at the earliest convenience for the patient. This will be in a private room and will outline the benefits of stopping smoking before thoracic surgery, discuss pharmacotherapy options, provide behavioural support (25-28), decide on an early quit date (aim within 48 hours of consent) and provide further information regarding *'Quit4Surgery'*. The HCP will validate and quantify smoking amount using CO measurements, which will be repeated at subsequent face-to-face interactions, providing biofeedback upon the success of smoking cessation to the patient.

For peer review only - http://bmjopen.bmj.com/site/about/guidelines.xhtml

2) Pharmacotherapy is encouraged for all patients and includes either combined short- and long-acting nicotine replacement therapy (NRT) or varenicline, which are provided as standard care within the NHS. Patients wishing to use e-cigarettes will be given advice as per the NCSCT guidance (29). Treatment will be maintained for the peri-operative period to offset nicotine withdrawal symptoms and is typically 8-12 weeks for NRT and 12 weeks for varenicline as per NCSCT guidance, with pharmacotherapy and behavioural support tailored appropriately to individual patients.

3) *'Quit4Surgery'* will collect feedback regarding the patient's engagement, cravings and abstinence and provide motivational feedback to support the patient. The motivational feedback provided by the web app is guided by established behavioural change theory and will complement the patients smoking cessation package. Patient feedback using the web app to the surgical team will guide them as to the need for additional contact if the patient wishes. This will help improve overall efficacy of the intervention.

INT patients will receive proactive support within 2 days of the quit date (in person or by telephone) and then weekly until one month after surgery. The weekly sessions will concentrate on facilitators and barriers to quitting, relapse, pharmacotherapy side effects and withdrawal symptoms. Face-to-face interactions will occur during the surgical outpatient appointments, pre-clerking clinic and during hospital admission to reduce the number of additional visits. Thus the INT will fit into the 'referral to treatment' target time frame and ensuring surgery is not delayed as recommended by NICE.

Control group of usual care (UC)

Termed usual care (UC), per usual local practice, patients are typically given a leaflet about the benefits of smoking cessation and referred to their local NHS smoking cessation service, which typically last up to 12 weeks and include pharmacotherapy. Both groups will receive the same pre-, peri- and post-operative care as per protocol.

Withdrawal from the Trial

Withdrawal from the trial before surgery is a decision of the participant. However participants will be asked if the research team can still use the data collected during their participation in the research analyses.

For peer review only - http://bmjopen.bmj.com/site/about/guidelines.xhtml

Protocol Deviations

All study and protocol deviations will be documented in the patients CRF and reported to the Principle Investigator, who will notify the Sponsor of any serious breaches. Patients will be analysed according to group allocation, by intent-to-treat analysis.

Patient and Public Involvement

The project details were discussed at a national thoracic surgery patient group (RESOLVE) meeting and feedback regarding merit and acceptability of the proposed intervention were incorporated. Dissemination of results will occur via specific patient feedback events. A patient representative was an active contributor to the development of the trial and intervention and will be a member of the trial management group. The intervention including the web app *'Quit4Surgery'* were designed to meet patients' specific 'needs' and 'drivers' of smokers who are waiting for thoracic surgery, with a PPI group involved in the design and initial product testing stages of this feasibility study.

OUTCOME AND DATA COLLECTION

Patient Recruitment into study

The overall aims of the feasibility are to find out if a larger trial is feasible. The quantitative measurements related to this include:

- Proportion of all elective thoracic procedures screened
- Proportion of eligible participants of those screened
- Proportion of eligible participants consented to receive intervention

In this feasibility study, selecting 5 units whose overall elective thoracic patient through-put amounts to 4000 patients a year, of whom 20% are smokers and 20% agree to take part in the study. It is expected that 60 eligible patients will be recruited to the INT group and 60 patients to the UC group of the study. Therefore the aim is to recruit 5 patients a month to each group over the 12 months recruitment period across all sites.

Patient Identification and Screening

For peer review only - http://bmjopen.bmj.com/site/about/guidelines.xhtml

The proportion of patients screened for eligibility and recorded on a screening log will be assessed and reported as the proportion of patients screened from the total number of planned major thoracic surgery during the study period.

Reasons for Failure to be Recruited

The proportion of patients that were missed, which should be minimal and proportion of patients who decline to take part will be recorded. Patients decline participation for many reasons, which should be captured whenever possible.

Education Material of Nurses and Surgeons

Feedback on the appropriateness, value and acceptability of the training will be elicited to enable refinement of the training programme for the substantive study, and to define a minimum competence. The training material will be evaluated for its ease of use, should it be used in the substantive study.

Assessment of Data Collection Process

Data will be collected using a CRF and will include demographic information and co-morbidities. Postoperative complications will be defined by the European Society of Thoracic Surgery (ESTS) (30), Thoracic Morbidity and Mortality (TM&M) system (31) and the stEP-COMPAC Group (32); see online supplementary appendix for definitions. Hospital re-admission rate will be determined within 30 days of discharge.

Assessment and identification will be made for loss of data during in-hospital stay to improve the data collection process for the substantive trial.

Smoking-related Outcomes

In the study the feasibility of the following questionnaires will be tested:

- Self-reported quit rate and exhaled CO measurement
- Fagerstrom Test for Nicotine Dependency: assessment of nicotine addiction (33).
- Mood and Physical Symptoms Scale (MPSS): assessment of cigarette withdrawal symptoms over the past 24 hours, including the strength of urge to smoke (34).

For peer review only - http://bmjopen.bmj.com/site/about/guidelines.xhtml
For peer review only

- Generic health related quality of life (EQ-5D-5L): assessment to provide a preference-based measure of health-related quality-of-life (35).
- NRT and support usage questionnaire.
- Health resource usage questionnaire: assessment of resource will be assessed via patient-recall with resources being measured including planned hospital overnight stays, planned hospital out-patient visits, hospital emergency visits, hospital admissions, GP and other community service visits.

Acceptability to and Impact on Patients

All patients consenting to participate in the trial will be eligible for interview and selected using maximum variety sampling by age, sex, ethnicity, admitting diagnosis, surgical procedure and smoking status. Interviews will be conducted until saturation is achieved, which is likely to be around 25-30 patients across all sites (36). Telephone interviews will be conducted as to minimise impact to patients following major surgery, will last no longer than 60 minutes and will be audio recorded and transcribed by the researcher.

An interview guide will be developed using evidence from previous experience of running the rehabilitation and pain study, and based on the interview objectives: pre- and post-surgical experiences of patients receiving the intervention (including effectiveness of staff communication) and patient engagement with it, any unintended consequences, acceptability of the intervention to patients, and recommendations for how its use or content/design could be improved.

Assessment of Trial Processes and Impact on Staff

Key HCPs in the clinical pathway will be invited to attend a focus group or individual interviews that will explore both the acceptability and recommendations for optimisation of the intervention.

Digitally recorded interviews and focus groups will be transcribed verbatim and anonymised. Transcripts will be analysed for patients and staff separately following Braun and Clarke's method for thematic analysis. Analysis will take an iterative approach, where data collection and analysis occurs concurrently, allowing the topic guide to be modified throughout to reflect emergent and/or priority themes.

For peer review only - http://bmjopen.bmj.com/site/about/guidelines.xhtml

STATISTICS AND DATA COLLECTION

Sample Size Calculation

As this is a feasibility study no formal sample size calculation has been performed. An audit discussing sample size in pilot and feasibility studies concluded that while sample size justification is important, formal calculation may not be appropriate. The findings from the audit concluded that a median size of 30 in each arm is appropriate (37). The study will aim to enrol 60 participants in the intervention over 1 year as this is a sufficient number to estimate a proportion of patients who have quit smoking by the day of surgery (38), as well as to explore data collection processes, and inform sample size calculations for a potential larger trial. 60 participants will allow us to measure recruitment and compliance rates with 95% confidence interval (CI) width between 10 and 20%. It would also be enough to estimate the standard deviation (SD) of questionnaires with reasonable accuracy for future planning of a larger trial.

Data Analysis

Appropriate summary statistics (e.g. proportions and inter-quartile ranges, means and SDs) will be generated for the study feasibility and patient/clinical measures. Between-group measures (mean differences, etc.) will be generated alongside 95% CI, however formal hypothesis testing will not be carried out as the aim here is not to conclusively prove efficacy and furthermore the size of sample is too small for any inferential tests to be meaningful. Participants will be kept in the groups they were allocated, regardless of compliance with treatment (intention-to-treat protecting against attrition bias). Analysis will be completed once all participants have completed all follow-up assessments. All data used in publication will be in an anonymous format in order to maintain patient study participation confidential.

Handling Missing Data

A member of the research team will contact patients for any missing data (e.g. questionnaires) via telephone and post. Where patients attend for follow-up clinic, the potential for missing data will again be limited. Imputation of missing responses is not proposed for patient reported outcomes as this is not a definitive trial and no hypothesis testing will be performed.

For peer review only

DATA MANAGEMENT AND QUALITY ASSURANCE

Data management and confidentiality

Personal data will be collected from trial participants and hospital notes on CRFs, coded with the participant's unique trial number and initials. This will be held securely and strictly confidentially according to NHS policies. Patients in the semi-structured qualitative interviews will be consented specifically for their name, address and contact telephone number to be shared with University of Birmingham (UoB) and University College London. Data will be transferred securely by encrypted end-to-end email and will not be labelled with private identifiable information. Interview response information will be kept encrypted on a computer in a locked office. No data that could be used to identify an individual will be published. Data will be stored on a secure server under the provisions of the Data Protection Act and/or applicable laws and regulations. Data may be accessed by external regulatory agencies and the Study Sponsor representatives and permission for this access will be documented within the participants consent form.

Monitoring and Audit

Onsite monitoring will be conducted as required by the UoB Clinical Research Compliance Team, with activities reported to the trials team and any issues noted followed-up to resolution. Additional on-site monitoring visits may be triggered, e.g. by poor CRF return, poor data quality, low AE reporting rates, excessive number of participant withdrawals/deviations. Study data and evidence of monitoring and systems audits will be made available for inspection by the regulatory authority as required.

Long Term Storage of Data

Trial data will be stored archived after the formal closure of the trial in accordance with archive policy and for the appropriate duration as per current legislation.

Data access

Upon completion and publication of the study, individual participant data will be shared that underlie the results reported in study after de-identification. Additional related documents will

For peer review only - http://bmjopen.bmj.com/site/about/guidelines.xhtml

be available including the study protocol, statistical analysis plan an analytical code. This data will be available in the beginning 3 months and 5 years following article publication to those who provide a methodologically sound proposal for analysis to achieve the aims in the approved proposal. All proposals should be directed to b.naidu@bham.ac.uk. To gain access requestors will need to sign a data access agreement. Data will be available for 5 years at a third party website.

SPONSORSHIP AND INDEMNITY

The UoB will act as the sponsor for this study. Delegated responsibilities will be assigned to the Chief Investigator and the NHS Trusts involved in the study. The UoB has in force a Public Liability Policy and/or Clinical Trials policy which provides cover for claims for 'negligent harm' and the activities here are included within that coverage.

REGULATORY APPROVALS

This study has obtained ethical approval from the NHS West Midlands Black Country Research Ethics Committee (Protocol Version 2.1; REC number 19/WM/0097).

STUDY DISSEMINATION

This aim of this feasibility study is to inform a substantive trial. On completion, results will be published in a peer-review scientific journal. The feasibility study is registered on the clinical trial database (NCT04190966).

Author Contributions: All authors have made substantial contributions to the conception or design of the work, or acquisition, analysis or interpretation of the data. STL has been participated in the study design, intervention development and drafted the manuscript. SK, AK and AB have participated in the intervention development. AF, OP, JB, RW, DRT, and BN have participated in the study design, intervention development and have critically revised the manuscript. All authors have approved the final version.

Funding: The research costs of the trial are funded by a grant from Johnson and Johnson

Competing Interests: None declared

Ethics approval: NHS Research Ethics Committee (REC number 19/WM/0097)

For peer review only - http://bmjopen.bmj.com/site/about/guidelines.xhtml

REFERENCES

1. 2017 [Available from: https://scts.org/wp-content/uploads/2017/04/National-2014-15-returns-for-SCTS_org-corrected-11-04-2017.xlsx.
2. Agostini P, Lugg ST, Adams K, Vartsaba N, Kalkat MS, Rajesh PB, et al. Postoperative pulmonary complications and rehabilitation requirements following lobectomy: a propensity score matched study of patients undergoing video-assisted thoracoscopic surgery versus thoracotomydagger. Interactive cardiovascular and thoracic surgery. 2017;24(6):931-7.
3. Agostini P, Cieslik H, Rathinam S, Bishay E, Kalkat MS, Rajesh PB, et al. Postoperative pulmonary complications following thoracic surgery: are there any modifiable risk factors? Thorax. 2010;65(9):815-8.
4. Lugg ST, Tikka T, Agostini PJ, Kerr A, Adams K, Kalkat MS, et al. Smoking and timing of cessation on postoperative pulmonary complications after curative-intent lung cancer surgery. Journal of cardiothoracic surgery. 2017;12(1):52.
5. Parsons A, Daley A, Begh R, Aveyard P. Influence of smoking cessation after diagnosis of early stage lung cancer on prognosis: systematic review of observational studies with meta-analysis. BMJ (Clinical research ed). 2010;340:b5569.
6. NICE Guidance smoking cessation in secondary care acute maternity and mental health services.
7. Thomsen T, Villebro N, Moller AM. Interventions for preoperative smoking cessation. The Cochrane database of systematic reviews. 2014(3):Cd002294.
8. Moller AM, Villebro N, Pedersen T, Tonnesen H. Effect of preoperative smoking intervention on postoperative complications: a randomised clinical trial. Lancet (London, England). 2002;359(9301):114-7.
9. Lindstrom D, Sadr Azodi O, Wladis A, Tonnesen H, Linder S, Nasell H, et al. Effects of a perioperative smoking cessation intervention on postoperative complications: a randomized trial. Ann Surg. 2008;248(5):739-45.
10. Webb J KA, Thickett D, Naidu B. Smoking habits of pre-surgery patients. . European Respiratory Journal. 2015;46: PA2511.
11. Warner DO, Klesges RC, Dale LC, Offord KP, Schroeder DR, Vickers KS, et al. Telephone quitlines to help surgical patients quit smoking patient and provider attitudes. American journal of preventive medicine. 2008;35(6 Suppl):S486-93.
12. Lee SM, Landry J, Jones PM, Buhrmann O, Morley-Forster P. Long-term quit rates after a perioperative smoking cessation randomized controlled trial. Anesthesia and analgesia. 2015;120(3):582-7.
13. Farley A, Aveyard P, Kerr A, Naidu B, Dowswell G. Surgical lung cancer patients' views about smoking and support to quit after diagnosis: a qualitative study. Journal of cancer survivorship : research and practice. 2016;10(2):312-9.
14. Park ER, Japuntich S, Temel J, Lanuti M, Pandiscio J, Hilgenberg J, et al. A smoking cessation intervention for thoracic surgery and oncology clinics: a pilot trial. Journal of thoracic oncology : official publication of the International Association for the Study of Lung Cancer. 2011;6(6):1059-65.
15. NICE. Lung cancer: diagnosis and management. NICE guideline [CG121] 2011 [Available from: https://www.nice.org.uk/guidance/CG121.
16. National Cancer Waiting Times Monitoring Dataset Guidance – Version 9.0. 2015.
17. Thornton L, Quinn C, Birrell L, Guillaumier A, Shaw B, Forbes E, et al. Free smoking cessation mobile apps available in Australia: a quality review and content analysis. Australian and New Zealand journal of public health. 2017;41(6):625-30.
18. McBride CM, Ostroff JS. Teachable moments for promoting smoking cessation: the context of cancer care and survivorship. Cancer control : journal of the Moffitt Cancer Center. 2003;10(4):325-33.

For peer review only - http://bmjopen.bmj.com/site/about/guidelines.xhtml

19. Shi Y, Warner DO. Surgery as a teachable moment for smoking cessation. Anesthesiology. 2010;112(1):102-7.
20. Hemming K, Haines TP, Chilton PJ, Girling AJ, Lilford RJ. The stepped wedge cluster randomised trial: rationale, design, analysis, and reporting. BMJ (Clinical research ed). 2015;350:h391.
21. What is Scrum? An Agile Framework for Completing Complex Projects - Scrum Alliance. Scrum Alliance.2016.
22. A M. Strategy, Implementation, Organisation and People. Springer Verlag2015.
23. Michie S, Hyder N, Walia A, West R. Development of a taxonomy of behaviour change techniques used in individual behavioural support for smoking cessation. Addict Behav. 2011;36(4):315-9.
24. Standard Treatment Programme 2012 [Available from: http://www.ncsct.co.uk/usr/pub/NCSCT%20STP.pdf.
25. West R, Sohal T. "Catastrophic" pathways to smoking cessation: findings from national survey. BMJ (Clinical research ed). 2006;332(7539):458-60.
26. Ong KC, Cheong GN, Prabhakaran L, Earnest A. Predictors of success in smoking cessation among hospitalized patients. Respirology (Carlton, Vic). 2005;10(1):63-9.
27. Sivarajan Froelicher ES, Miller NH, Christopherson DJ, Martin K, Parker KM, Amonetti M, et al. High rates of sustained smoking cessation in women hospitalized with cardiovascular disease: the Women's Initiative for Nonsmoking (WINS). Circulation. 2004;109(5):587-93.
28. BTS Recommendations for smoking cessation services.
29. NCSCT. Electronic Cigarettes - A briefing for stop smoking services. 2016.
30. (ESTS) ESoTS. Database report silver book 2015. Available online: . 2015.
31. Seely AJ, Ivanovic J, Threader J, Al-Hussaini A, Al-Shehab D, Ramsay T, et al. Systematic classification of morbidity and mortality after thoracic surgery. The Annals of thoracic surgery. 2010;90(3):936-42; discussion 42.
32. Abbott TEF, Fowler AJ, Pelosi P, Gama de Abreu M, Moller AM, Canet J, et al. A systematic review and consensus definitions for standardised end-points in perioperative medicine: pulmonary complications. British journal of anaesthesia. 2018;120(5):1066-79.
33. Heatherton TF, Kozlowski LT, Frecker RC, Fagerstrom KO. The Fagerstrom Test for Nicotine Dependence: a revision of the Fagerstrom Tolerance Questionnaire. British journal of addiction. 1991;86(9):1119-27.
34. West R, Hajek P. Evaluation of the mood and physical symptoms scale (MPSS) to assess cigarette withdrawal. Psychopharmacology. 2004;177(1-2):195-9.
35. EuroQol--a new facility for the measurement of health-related quality of life. Health policy (Amsterdam, Netherlands). 1990;16(3):199-208.
36. Morse JM. Analytic Strategies and Sample Size. Qualitative health research. 2015;25(10):1317-8.
37. Billingham SA, Whitehead AL, Julious SA. An audit of sample sizes for pilot and feasibility trials being undertaken in the United Kingdom registered in the United Kingdom Clinical Research Network database. BMC medical research methodology. 2013;13:104.
38. Browne RH. On the use of a pilot sample for sample size determination. Statistics in medicine. 1995;14(17):1933-40.

For peer review only

For peer review only - http://bmjopen.bmj.com/site/about/guidelines.xhtml

Table 1 Summary of investigations and assessments

	**Participants**		**Time points**					
	**Intervention Group (INT)**	**Usual Care Group (UC)**	**Baseline clinic prior to surgery**	**Day of surgery**	**During hospital Stay**	**Day of hospital discharge**	**One month after surgery**	**Six months after enrolment**
Eligibility and written informed consent	*X*	*X*	*X*					
Demographic Data[1]	*X*	*X*	*X*					
Previous Medical History[2]	*X*	*X*	*X*					
Self-reported Quit rate	*X*	*X*	*X*	*X*		*X*	*X*	*X*
Exhaled CO measurement	*X*	*X*	*X*	*X*		*X*	*X*	*X*
NRT and support usage questionnaire	*X*	*X*	*X*	*X*		*X*	*X*	*X*
Fagerstrom Test for Nicotine Dependence	*X*	*X*	*X*				*X*	
Mood and Physical Symptoms Scale	*X*		*X*	*X*		*X*	*X*	
EQ-5D-5L	*X*		*X*				*X*	
Health resource usage questionnaire	*X*						*X*	
Surgery and anesthetic data[3]	*X*	*X*		*X*				
Postoperative complications[4,5]	*X*	*X*			*X*	*X*	*X*	
Hospital readmission[6]	*X*	*X*					*X*	
Semi structured qualitative interviews[7]	*X*	*X*					*X*	
Adverse events	*If applicable*							
Protocol deviations	*If applicable*							

1) Demographic data: gender, age, indication for surgery, height, weight, BMI, ASA grade, ECOG score, dyspnoea score, recent lung function
2) Previous medical history: smoking history, alcohol intake per week, co-morbidities (COPD, Ischaemic Heart Disease, Congestive Cardiac Failure, Hypertension, diabetes (diet-controlled/ oral therapy/ insulin), renal failure, previous stroke, thyroid disease (hyperthyroid/ hypothyroid).
3) Anaesthetic technique data: PCA, Epidural, Paravertebral and morphine infusion, overall fluid balance, blood loss, operation performed (side of surgery, operation, surgical technique).
4) **Post-operative data and observations**: Routine blood results if done (full blood count, albumin, renal function, electrolytes, CRP)
   Acute complications: According to ESTS (30) (Appendix A) and Thoracic Morbidity and Mortality Classification (31) (Appendix B), data also collected including admission and

For peer review only

For peer review only - http://bmjopen.bmj.com/site/about/guidelines.xhtml

length of stay on the ward (0), step-down (1), the HDU (2) and ITU (3). Data will also be collected in patients requiring mini-tracheostomy or additional surgery.

Post-operative pulmonary complications: Using stEP-COMPAC Group definition of postoperative pulmonary complications (32) (Appendix C) defining atelectasis (detected on computer tomography/CXR), pneumonia (using US Centres for Disease Control criteria), acute respiratory distress syndrome (using Berlin Consensus), and pulmonary aspiration (clear clinical history and radiological evidence).

5) Discharge data: total hospital stay, home with flutter bag, histology data and mortality.
6) Follow-up: hospital readmission up to and including one month following surgery.
7) At 4-8 weeks post-surgery patients will also have semi-structured qualitative patient interviews will be undertaken at 4 weeks post discharge to investigate experience, engagement, acceptability, unintended consequences/benefits and how to optimise the intervention delivery.

For peer review only

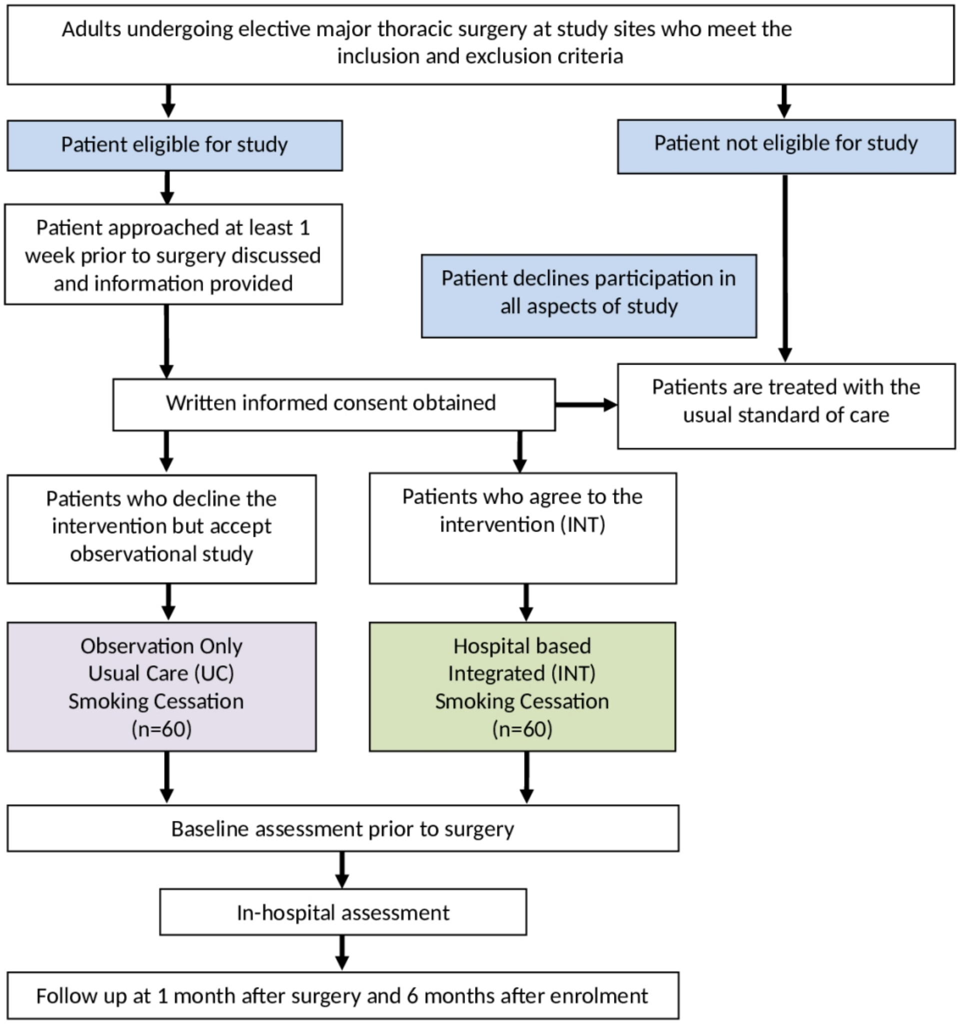

Trial Schema

168x179mm (200 x 200 DPI)

APPENDIX

Appendix A – Major cardiopulmonary complications as classified by the European Society of Thoracic Surgeons (ESTS)

ARDS: Adult respiratory distress syndrome defined according to the American-European consensus conference. All of the following criteria should be met:
1. Acute onset
2. Arterial hypoxemia with PaO2/FIO2 ratio lower than 200 (regardless PEEP level)
3. Bilateral infiltrates at chest radiograph or CT scan
4. No clinical evidence of left atrial hypertension or pulmonary artery occlusive pressure <18 mmHg
5. Compatible risk factors

Atrial Arrhythmia: new onset of atrial fibrillation/flutter (AF) requiring medical treatment or cardioversion. Does not include recurrence of AF which had been present preoperatively.

Ventricular Arrhythmia: sustained ventricular tachycardia or ventricular fibrillation that has been clinically documented and treated by ablation therapy, implantable cardioverter defibrillator, permanent pacemaker, pharmacologic treatment or cardioversion.

Bronchoscopy for atelectasis: postoperative atelectasis documented clinically or radiographically that needed bronchoscopy.

Pneumonia: defined according to the last CDC criteria. Two or more serial chest radiographs with at least **one** of the following:

- New or progressive and persistent infiltrate
- Consolidation
- Cavitation

AND at least **one** of the following:

- Fever (>38°C or >100.4°F) with no other recognized cause
- Leukopenia (<4000 WBC/mm3) or leukocytosis (>12,000 WBC/mm3)
- For adults >70 years old, altered mental status with no other recognized cause

AND at least **two** of the following:

- New onset of purulent sputum, or change in character of sputum, or increased respiratory secretions, or increased suctioning requirements

For peer review only

- New onset or worsening cough, or dyspnea, or tachypnea
- Rales or bronchial breath sounds Worsening gas exchange (e.g. O2 desaturations (e.g., PaO2/FiO2 < 240), increased oxygen requirements, or increased ventilator demand).

Pulmonary embolism: confirmed by V/Q scan, angiogram or CT scan.

DVT: deep venous thrombosis confirmed by Doppler study, contrast study or other study and that required treatment.

Myocardial infarct: evidenced by one of the following criteria:

1. Transmural infarction diagnosed by the appearance of a new Q wave in two or more contiguous leads on ECG.
2. Subendocardial infarction (non Q wave) evidenced by clinical, angiographic electrocardiographic signs.
3. Laboratory isoenzyme evidence of myocardial necrosis.

Renal failure: defined as the onset of new renal failure in the postoperative period according to one of the following criteria:

1. Increase of serum creatinine to greater than 2.0, and 2-fold the preoperative creatinine level.
2. A new requirement for dialysis postoperatively.

Neurological complication: occurrence of one of the following central neurologic postoperative events not present preoperatively:

1. A central neurologic deficit persisting postoperatively for more than 72 hours
2. A transient neurologic deficit (transient ischemic attack or reversible ischemic neurological deficit) with recovery within 72 hours
3. A new postoperative coma persisting at least 24 hours and caused by anoxic/ischemic and/or metabolic encephalopathy, thromboembolic event or cerebral bleed

For peer review only

Appendix B – Seeley Systematic Classification of Morbidity and Mortality After Thoracic Surgery (TM &M) Classification of Severity

Complication: Any deviation from the normal postoperative course.

**Minor**	
Grade I	Any complication without need for pharmacologic treatment or other intervention.
Grade II	Any complication that requires pharmacologic treatment or minor intervention only.
**Major**	
Grade III	Any complication that requires surgical, radiologic, endoscopic intervention, or multi-therapy.
Grade IIIa	Intervention does not require general anaesthesia.
Grade IIIb	Intervention requires general anaesthesia.
Grade IV	Any complication requiring intensive care unit management and life support.
Grade IVa	Single organ dysfunction.
Grade IVb	Multi-organ dysfunction.
**Mortality**	
Grade V	Any complication leading to the death of the patient.

For peer review only

Appendix C – StEP Core Outcome Measures in Perioperative and Anaesthetic Care (COMPAC) – Post-operative Pulmonary Complications

Post-operative Pulmonary Complications*

Composite of respiratory diagnoses that share common pathophysiological mechanisms including pulmonary collapse and airway contamination:

(i) atelectasis detected on computed tomography or chest radiograph,

(ii) pneumonia using US Centers for Disease Control criteria,

(iii) Acute Respiratory Distress Syndrome using Berlin consensus definition,

(iv) pulmonary aspiration (clear clinical history ***AND*** radiological evidence).

***Exclusions**

Other diagnoses that do not share a common biological mechanism are best evaluated separately and only when clearly relevant to the treatment under investigation:

(i) pulmonary embolism,

(ii) pleural effusion,

(iii) cardiogenic pulmonary oedema,

(iv) pneumothorax,

(v) bronchospasm.

ARDS - Berlin definition

Timing: within 1 week of a known clinical insult or new or worsening respiratory symptoms

AND Chest imaging: bilateral opacities not fully explained by effusions, lobar/lung collapse or nodules

AND Origin of oedema: respiratory failure not fully explained by cardiac failure or fluid overload (requires objective assessment, e.g. echocardiography, to exclude hydrostatic oedema),

AND Oxygenation:

Mild PaO_2:FiO_2 between 26.7 - 40.0 kPa (200-300 mm Hg) with PEEP or CPAP $\geq$ 5 cm H_2O;

Moderate PaO_2:FiO_2 between 13.3 - 26.6 kPa (100-200 mm Hg) with PEEP $\geq$ 5 cmH_2O;

Severe PaO_2:FiO_2 $\leq$ 13.3 kPa (100 mm Hg) with PEEP $\geq$ 5 cm H_2O.

Mechanical ventilation:

The need for need for tracheal re-intubation and mechanical ventilation after extubation, and within 30 days after surgery OR mechanical ventilation for more than 24 h after surgery. The inclusion of non-invasive ventilation may be considered on a study-by-study basis.

For peer review only

Post-operative Pneumonia

Two or more serial chest radiographs with at least one of the following (one radiograph is sufficient for patients with no underlying pulmonary or cardiac disease):

(i) New or progressive and persistent infiltrates,

(ii) consolidation

(iii) cavitation;

AND at least **one** of the following:

(a) fever (>38°C) with no other recognised cause,

(b) leucopaenia (white cell count <4x10^{9}/1) or leucocytosis (white cell count >12x10^{9}/l),

(c) for adults >70 years old, altered mental status with no other recognised cause;

AND at least **two** of the following:

(a) new onset of purulent sputum or change in character of sputum, or increased respiratory secretions, or increased suctioning requirements,

(b) new onset or worsening cough, or dyspnoea, or tachypnoea,

(c) rales or bronchial breath sounds,

(d) worsening gas exchange (hypoxaemia, increased oxygen requirement, increased ventilator demand).

For peer review only

Reporting checklist for protocol of a clinical trial.

Based on the SPIRIT guidelines.

Instructions to authors

Complete this checklist by entering the page numbers from your manuscript where readers will find each of the items listed below.

Your article may not currently address all the items on the checklist. Please modify your text to include the missing information. If you are certain that an item does not apply, please write "n/a" and provide a short explanation.

Upload your completed checklist as an extra file when you submit to a journal.

In your methods section, say that you used the SPIRITreporting guidelines, and cite them as:

Chan A-W, Tetzlaff JM, Altman DG, Laupacis A, Gøtzsche PC, Krleža-Jerić K, Hróbjartsson A, Mann H, Dickersin K, Berlin J, Doré C, Parulekar W, Summerskill W, Groves T, Schulz K, Sox H, Rockhold FW, Rennie D, Moher D. SPIRIT 2013 Statement: Defining standard protocol items for clinical trials. Ann Intern Med. 2013;158(3):200-207

		Reporting Item	Page Number
**Administrative information**			
Title	#1	Descriptive title identifying the study design, population, interventions, and, if applicable, trial acronym	1
Trial registration	#2a	Trial identifier and registry name. If not yet registered, name of intended registry	1
Trial registration: data set	#2b	All items from the World Health Organization Trial Registration Data Set	1
Protocol version	#3	Date and version identifier	16
Funding	#4	Sources and types of financial, material, and other support	16
Roles and responsibilities: contributorship	#5a	Names, affiliations, and roles of protocol contributors	16

For peer review only

Roles and responsibilities: sponsor contact information	#5b	Name and contact information for the trial sponsor	1
Roles and responsibilities: sponsor and funder	#5c	Role of study sponsor and funders, if any, in study design; collection, management, analysis, and interpretation of data; writing of the report; and the decision to submit the report for publication, including whether they will have ultimate authority over any of these activities	16
Roles and responsibilities: committees	#5d	Composition, roles, and responsibilities of the coordinating centre, steering committee, endpoint adjudication committee, data management team, and other individuals or groups overseeing the trial, if applicable (see Item 21a for data monitoring committee)	16
**Introduction**			
Background and rationale	#6a	Description of research question and justification for undertaking the trial, including summary of relevant studies (published and unpublished) examining benefits and harms for each intervention	4
Background and rationale: choice of comparators	#6b	Explanation for choice of comparators	5
Objectives	#7	Specific objectives or hypotheses	6
Trial design	#8	Description of trial design including type of trial (eg, parallel group, crossover, factorial, single group), allocation ratio, and framework (eg, superiority, equivalence, non-inferiority, exploratory)	7
**Methods: Participants, interventions, and outcomes**			
Study setting	#9	Description of study settings (eg, community clinic, academic hospital) and list of countries where data will be collected. Reference to where list of study sites can be	7

For peer review only

		obtained	
Eligibility criteria	#10	Inclusion and exclusion criteria for participants. If applicable, eligibility criteria for study centres and individuals who will perform the interventions (eg, surgeons, psychotherapists)	8
Interventions: description	#11a	Interventions for each group with sufficient detail to allow replication, including how and when they will be administered	9
Interventions: modifications	#11b	Criteria for discontinuing or modifying allocated interventions for a given trial participant (eg, drug dose change in response to harms, participant request, or improving / worsening disease)	9
Interventions: adherance	#11c	Strategies to improve adherence to intervention protocols, and any procedures for monitoring adherence (eg, drug tablet return; laboratory tests)	9
Interventions: concomitant care	#11d	Relevant concomitant care and interventions that are permitted or prohibited during the trial	9
Outcomes	#12	Primary, secondary, and other outcomes, including the specific measurement variable (eg, systolic blood pressure), analysis metric (eg, change from baseline, final value, time to event), method of aggregation (eg, median, proportion), and time point for each outcome. Explanation of the clinical relevance of chosen efficacy and harm outcomes is strongly recommended	7
Participant timeline	#13	Time schedule of enrolment, interventions (including any run-ins and washouts), assessments, and visits for participants. A schematic diagram is highly recommended (see Figure)	7
Sample size	#14	Estimated number of participants needed to achieve study objectives and how it was determined, including clinical and statistical assumptions supporting any sample size calculations	14
Recruitment	#15	Strategies for achieving adequate participant enrolment to reach target sample size	11

For peer review only

**Methods: Assignment of interventions (for controlled trials)**			
Allocation: sequence generation	#16a	Method of generating the allocation sequence (eg, computer-generated random numbers), and list of any factors for stratification. To reduce predictability of a random sequence, details of any planned restriction (eg, blocking) should be provided in a separate document that is unavailable to those who enrol participants or assign interventions	n/a
Allocation concealment mechanism	#16b	Mechanism of implementing the allocation sequence (eg, central telephone; sequentially numbered, opaque, sealed envelopes), describing any steps to conceal the sequence until interventions are assigned	n/a
Allocation: implementation	#16c	Who will generate the allocation sequence, who will enrol participants, and who will assign participants to interventions	n/a
Blinding (masking)	#17a	Who will be blinded after assignment to interventions (eg, trial participants, care providers, outcome assessors, data analysts), and how	n/a
Blinding (masking): emergency unblinding	#17b	If blinded, circumstances under which unblinding is permissible, and procedure for revealing a participant’s allocated intervention during the trial	n/a
**Methods: Data collection, management, and analysis**			
Data collection plan	#18a	Plans for assessment and collection of outcome, baseline, and other trial data, including any related processes to promote data quality (eg, duplicate measurements, training of assessors) and a description of study instruments (eg, questionnaires, laboratory tests) along with their reliability and validity, if known. Reference to where data collection forms can be found, if not in the	12

For peer review only

		protocol	
Data collection plan: retention	#18b	Plans to promote participant retention and complete follow-up, including list of any outcome data to be collected for participants who discontinue or deviate from intervention protocols	14
Data management	#19	Plans for data entry, coding, security, and storage, including any related processes to promote data quality (eg, double data entry; range checks for data values). Reference to where details of data management procedures can be found, if not in the protocol	15
Statistics: outcomes	#20a	Statistical methods for analysing primary and secondary outcomes. Reference to where other details of the statistical analysis plan can be found, if not in the protocol	14
Statistics: additional analyses	#20b	Methods for any additional analyses (eg, subgroup and adjusted analyses)	14
Statistics: analysis population and missing data	#20c	Definition of analysis population relating to protocol non-adherence (eg, as randomised analysis), and any statistical methods to handle missing data (eg, multiple imputation)	14
**Methods: Monitoring**			
Data monitoring: formal committee	#21a	Composition of data monitoring committee (DMC); summary of its role and reporting structure; statement of whether it is independent from the sponsor and competing interests; and reference to where further details about its charter can be found, if not in the protocol. Alternatively, an explanation of why a DMC is not needed	15
Data monitoring: interim analysis	#21b	Description of any interim analyses and stopping guidelines, including who will have access to these interim results and make the final decision to terminate the trial	15
Harms	#22	Plans for collecting, assessing, reporting, and managing solicited and spontaneously reported adverse events and other unintended effects of trial interventions or trial conduct	15
Auditing	#23	Frequency and procedures for auditing trial conduct, if	15

For peer review only

		any, and whether the process will be independent from investigators and the sponsor	
**Ethics and dissemination**			
Research ethics approval	#24	Plans for seeking research ethics committee / institutional review board (REC / IRB) approval	16
Protocol amendments	#25	Plans for communicating important protocol modifications (eg, changes to eligibility criteria, outcomes, analyses) to relevant parties (eg, investigators, REC / IRBs, trial participants, trial registries, journals, regulators)	16
Consent or assent	#26a	Who will obtain informed consent or assent from potential trial participants or authorised surrogates, and how (see Item 32)	15
Consent or assent: ancillary studies	#26b	Additional consent provisions for collection and use of participant data and biological specimens in ancillary studies, if applicable	15
Confidentiality	#27	How personal information about potential and enrolled participants will be collected, shared, and maintained in order to protect confidentiality before, during, and after the trial	15
Declaration of interests	#28	Financial and other competing interests for principal investigators for the overall trial and each study site	16
Data access	#29	Statement of who will have access to the final trial dataset, and disclosure of contractual agreements that limit such access for investigators	15
Ancillary and post trial care	#30	Provisions, if any, for ancillary and post-trial care, and for compensation to those who suffer harm from trial participation	16
Dissemination policy: trial results	#31a	Plans for investigators and sponsor to communicate trial results to participants, healthcare professionals, the public, and other relevant groups (eg, via publication, reporting in results databases, or other data sharing arrangements), including any publication restrictions	16
Dissemination policy:	#31b	Authorship eligibility guidelines and any intended use of	16

For peer review only

authorship		professional writers	
Dissemination policy: reproducible research	#31c	Plans, if any, for granting public access to the full protocol, participant-level dataset, and statistical code	16

Appendices

Informed consent materials	#32	Model consent form and other related documentation given to participants and authorised surrogates	n/a
Biological specimens	#33	Plans for collection, laboratory evaluation, and storage of biological specimens for genetic or molecular analysis in the current trial and for future use in ancillary studies, if applicable	n/a

The SPIRIT checklist is distributed under the terms of the Creative Commons Attribution License CC-BY-ND 3.0. This checklist was completed on 17. December 2019 using https://www.goodreports.org/, a tool made by the EQUATOR Network in collaboration with Penelope.ai

For peer review only

BMJ Open

Protocol for a feasibility study of smoking cessation in the surgical pathway before major lung surgery: Project MURRAY

Journal:	*BMJ Open*
Manuscript ID	bmjopen-2019-036568.R1
Article Type:	Protocol
Date Submitted by the Author:	29-Jun-2020
Complete List of Authors:	Lugg, Sebastian; University of Birmingham, Institute of Inflammation and Ageing<br>Kerr, Amy; University Hospitals Birmingham NHS Foundation Trust<br>Kadiri, Salma; University Hospitals Birmingham NHS Foundation Trust<br>Budacan, Alina; University Hospitals Birmingham NHS Foundation Trust<br>Farley, Amanda; University of Birmingham, Insitute of Applied Health Research<br>Perski, Olga; University College London, Institute of Epidemiology & Health<br>West, Robert; University College London, Institute of Epidemiology & Health<br>Brown, Jamie; University College London, Institute of Epidemiology & Health<br>Thickett , David ; University of Birmingham, Institute of Inflammation and Ageing<br>Naidu, Babu; University of Birmingham, Institute of Inflammation and Ageing
**Primary Subject Heading**:	Smoking and tobacco
Secondary Subject Heading:	Surgery, Respiratory medicine, Anaesthesia, Addiction
Keywords:	Cardiothoracic surgery < SURGERY, Adult thoracic medicine < THORACIC MEDICINE, Adult anaesthesia < ANAESTHETICS

SCHOLARONE™
Manuscripts

I, the Submitting Author has the right to grant and does grant on behalf of all authors of the Work (as defined in the below author licence), an exclusive licence and/or a non-exclusive licence for contributions from authors who are: i) UK Crown employees; ii) where BMJ has agreed a CC-BY licence shall apply, and/or iii) in accordance with the terms applicable for US Federal Government officers or employees acting as part of their official duties; on a worldwide, perpetual, irrevocable, royalty-free basis to BMJ Publishing Group Ltd ("BMJ") its licensees and where the relevant Journal is co-owned by BMJ to the co-owners of the Journal, to publish the Work in this journal and any other BMJ products and to exploit all rights, as set out in our licence.

The Submitting Author accepts and understands that any supply made under these terms is made by BMJ to the Submitting Author unless you are acting as an employee on behalf of your employer or a postgraduate student of an affiliated institution which is paying any applicable article publishing charge ("APC") for Open Access articles. Where the Submitting Author wishes to make the Work available on an Open Access basis (and intends to pay the relevant APC), the terms of reuse of such Open Access shall be governed by a Creative Commons licence – details of these licences and which Creative Commons licence will apply to this Work are set out in our licence referred to above.

Other than as permitted in any relevant BMJ Author's Self Archiving Policies, I confirm this Work has not been accepted for publication elsewhere, is not being considered for publication elsewhere and does not duplicate material already published. I confirm all authors consent to publication of this Work and authorise the granting of this licence.

For peer review only

TITLE

Protocol for a feasibility study of smoking cessation in the surgical pathway before major lung surgery: Project MURRAY

Corresponding author:

Mr Babu Naidu
Institute of Inflammation and Ageing, College of Medical and Dental Sciences,
University of Birmingham, B15 2TT
b.naidu@bham.ac.uk
Tel: +44 (0)121 371 3228

Sebastian T Lugg,[1,2] Amy Kerr,[1] Salma Kadiri,[1] Alina Budacan,[1] Amanda Farley,[3] Olga Perski,[4] Robert West,[4] Jamie Brown,[4] David Thickett,[1] Babu Naidu,[1] on behalf of the Project MURRAY Investigators

1. Institute of Inflammation and Ageing, College of Medical and Dental Sciences, University of Birmingham, Birmingham, UK.
2. Department of Thoracic Surgery, Heartlands Hospital, University Hospitals Birmingham NHS Foundation Trust, Birmingham, UK.
3. Institute of Applied Health Research, University of Birmingham, UK.
4. Institute of Epidemiology and Health, University College London, UK.

Keywords
Thoracic surgery, smoking cessation intervention, perioperative medicine, postoperative pulmonary complication, clinical trial

For peer review only

ABSTRACT

Introduction: Smoking prior to major thoracic surgery is the biggest risk factor for development of postoperative pulmonary complications, with 1 in 5 patients continuing to smoke before surgery. Current guidance is that all patients should stop smoking before elective surgery yet very few are offered specialist smoking cessation support. Patients would prefer support within the thoracic surgical pathway. No study has addressed the effectiveness of such an intervention in this setting on cessation. The overall aim is to determine in patients who undergo major elective thoracic surgery whether an intervention integrated (INT) into the surgical pathway improves smoking cessation rates compared with usual care (UC) of standard community/hospital based NHS smoking support. This pilot study will evaluate feasibility of a substantive trial.

Methods and analysis: Project MURRAY is a trial comparing the effectiveness of INT and UC on smoking cessation. INT is pharmacotherapy and a hybrid of behavioural support delivered by the trained healthcare practitioners (HCPs) in the thoracic surgical pathway and a complimentary web-based application. This pilot study will evaluate the feasibility of a substantive trial and study processes in 5 adult thoracic centres including the University Hospitals Birmingham NHS Foundation Trust. The primary objective is to establish the proportion of those eligible who agree to participate. Secondary objectives include evaluation of study processes. Analyses of feasibility and patient-reported outcomes will take the form of simple descriptive statistics and where appropriate, point estimates of effects sizes and associated 95% CIs.

Ethics and dissemination: The study has obtained ethical approval from NHS Research Ethics Committee (REC number 19/WM/0097). Dissemination plan includes: informing patients and HCPs; engaging multidisciplinary professionals to support a proposal of a definitive trial and submission for a full application dependent on the success of the study.

Trial Registration Number: NCT04190966; Pre-results

For peer review only - http://bmjopen.bmj.com/site/about/guidelines.xhtml
For peer review only

Strengths and limitations of the study

- This study addresses smoking in major thoracic surgery which can result in a significant economic and healthcare burden.
- This study will assess the role of a hybrid model of integrated smoking cessation within the surgical pathway delivered by trained healthcare practitioners and use of a web-based application.
- This feasibility study will assess patient recruitment to inform a definitive study.
- This study will add to the limited evidence towards of effective smoking cessation strategies in major thoracic surgery.
- This feasibility study will not answer the overarching research question of efficacy but will directly inform a well-designed definitive study.

For peer review only

For peer review only - http://bmjopen.bmj.com/site/about/guidelines.xhtml

INTRODUCTION

Poorer outcomes for smokers in thoracic surgery

25,000 patients undergo major thoracic surgery every year in the UK (1). 1 in 5 patients smoke before surgery, which increases the risk of developing postoperative pulmonary complications including pneumonia and lung collapse. Just under half of smokers develop these complications with associated increased in-hospital mortality (0.5% to 12%), intensive therapy unit admissions (1.5% to 26%), increased hospital stay (5 to 14 days) and poorer long-term outcomes (2-4). Furthermore, lung cancer surgery patients have an 86% increased risk for cancer recurrence and two-fold decrease in 5-year survival compared with patients who quit smoking at diagnosis (5).

The need for integrated hospital-based smoking cessation support

The current NICE guidance is that smokers undergoing ‘elective’ surgery should receive behavioural support and stop-smoking pharmacotherapy as early as possible in their outpatient or pre-operative assessments. This should be offered weekly, preferably face-to-face, for a minimum of 4 weeks after the quit date (6). The Cochrane review supporting this type of ‘intensive’ intervention (7) was based on two small trials in orthopaedics and general surgery (8, 9).

However in current practice most thoracic surgery patients do not receive any pre-operative smoking cessation support. Of the 120 patients attending a large UK thoracic regional unit only 40% of current smokers were offered support (10), similar to the proportion of all eligible pre-operative patients agreeing to participate in smoking cessation studies (11, 12). Only 1 in 3 smokers self-report abstinence at the time of lung cancer surgery (4), with biochemical verification indicating much lower quit rates (7).

Thus the current usual care (UC) of referral to stop-smoking services does not meet the standard set by NICE. This may be due to these services being designed to promote long-term quitting, which many smokers undergoing surgery may not be willing to commit to. Many patients also report that attending smoking cessation clinic appointments during their work-up for surgery is a significant barrier to stopping smoking and would prefer bespoke support during hospital visits (13).

For peer review only - http://bmjopen.bmj.com/site/about/guidelines.xhtml
For peer review only

In pre-operative smoking cessation studies, behavioural support is delivered either by research nurses or independent smoking cessation practitioners (12, 14). It is logical that HCPs in the surgical pathway could be trained to deliver the support due to the high prevalence of smoking within this patient group. This approach is advocated by both the Lung Cancer Nurse Forum and the Society of Cardiothoracic Surgery (UK) and evidence suggests that nurse delivered smoking cessation interventions are effective (15). Timely access is also crucial; NICE lung cancer guidelines recommend that surgery should not be delayed to give up smoking (16) and needs to be performed within 62 days of presentation and 31 days of diagnosis to avoid heavy financial penalties on individual Trusts (17). Therefore avoiding delays in receiving behavioural and pharmacological support is of paramount importance.

Poor quality of available smoking cessation applications

A combination of a web-based application (web app) with structured face-to-face behavioural support may aid successful quit attempts, whilst also supporting HCPs in delivering smoking cessation support throughout the surgical pathway. A recent review of over 100 smoking cessation apps showed that only 6 were deemed to be of high quality (18). There are no apps specifically designed for smoking cessation in patients undergoing major surgery and none designed to provide hybrid support alongside a trained smoking cessation practitioner.

Summary

Many patients who undergo thoracic surgery continue to smoke, and effective pre-operative smoking cessation interventions may improve outcomes. However, there are few studies exploring strategies for integrating smoking cessation into the patient pathway, which is a high priority area for research in these patients. We have developed a bespoke, tailored intense, integrated smoking cessation intervention to test in a 'real-life' clinical trial within the UK. The intervention involves pharmacotherapy and a hybrid of support delivered by trained HCPs in the thoracic surgical pathway and a complementary web app. Support may be particularly effective because of site of the surgery, the lungs, makes this a clear 'teachable moment' for patients (19, 20).

Study aim

For peer review only - http://bmjopen.bmj.com/site/about/guidelines.xhtml

The overall aim of this research is to determine if integrated (INT) smoking cessation support in the surgical pathway improves smoking cessation rates in patients undergoing major elective thoracic surgery when compared with usual care (UC) of standard community/hospital based NHS smoking cessation support. To answer this research question with substantial evidence of the clinical and cost-effectiveness of INT approach, a multi-centre RCT is required. Feasibility studies are a recommended pre-requisite to assess feasibility of a large and expensive full-scale trial. We have therefore designed this multicentre feasibility study, which aims evaluate the feasibility of the INT by making the following quantitative and qualitative assessments.

Objectives of the feasibility study

The aims of the feasibility study are to assess various aspects of the trial design and management and not to determine the relative effectiveness of INT versus UC.

Primary Objective

To establish the number of patients who agree to participate in the intervention as a proportion of those eligible to enter the study.

Secondary Objectives

1. Integration of the intervention into the clinical pathway by time from decision to operate from study recruitment.
2. Explore barriers to study recruitment, including descriptive reasons for non-participation from screening logs.
3. Fine-tune study procedures and pilot data capture forms aiming for over 90% completion of important perioperative data for each patient.
4. To assess the proportion of patients in the INT group who have quit smoking by the day of surgery and one month after surgery.
5. To assess the proportion of patients in the observation only UC group who have quit smoking by the day of surgery and one month after surgery.
6. To define the variability of smoking cessation practices in all patients using the nicotine replacement usage questionnaire.

For peer review only - http://bmjopen.bmj.com/site/about/guidelines.xhtml

7. Qualitative interview: To understand patients' experiences of and engagement with the intervention, and any unintended consequences; To establish whether the intervention is acceptable to thoracic surgery patients and staff and investigate recommendations for optimisation of intervention delivery.

TRIAL DESIGN

Design

Project Murray is an RCT comparing the effectiveness of INT verses UC in smoking cessation rates in patients undergoing major thoracic surgery, taking form of a stepped wedge cluster randomised controlled trial (21), and as such will not require individual patient randomisation. This feasibility study will evaluate the substantive trial and study processes.

Setting

Trial recruitment will be over a period of 12 months with an additional 6-month follow-up period. Recruitment will initiate at the University Hospitals Birmingham NHS Foundation Trust, which is the trial co-ordinating site and performs >1000 major thoracic surgical procedures a year. Additional recruitment will involve 4 further regional thoracic surgical centres performing >400 major thoracic surgical procedures a year.

Flow of participants during the trial

The anticipated pathway of patients through the trial is shown in the trial schema (Figure 1). All adult patients who fulfil the inclusion and exclusion criteria during the study period will be approached and participant information sheets (PIS) will be provided. Written informed consent will be obtained after an opportunity for patients to discuss requirements for the study. If the patient accepts the intervention, they will be placed into the INT group of the study. If they decline the intervention they will be invited to take part in the observational only part of the study and will receive usual care (UC). We will extract routinely collected pre- and post-operative data from patient's medical records and also collect data using questionnaires. Adverse events will be collected throughout the duration of the study. The summary of assessments is detailed in (Table 1).

For peer review only - http://bmjopen.bmj.com/site/about/guidelines.xhtml

For peer review only

Study Eligibility

Inclusion Criteria

- Current tobacco smoker (smoked within the last 28 days)
- Major Thoracic Surgery (including both open and minimally invasive approach)
- Able to provide written informed consent
- At least 1 weeks' time to surgery
- Age over 18 years

Exclusion criteria

- Emergency thoracic surgery
- Inability to perform exhaled carbon monoxide (CO) measurements

Patient Identification and screening procedure

Patients who are listed for major thoracic surgery will be identified and screened for eligibility prior to surgery. If a patient is screened and not eligible for the study or does not consent to be in the intervention or observation group, a record of the case will be kept in the screening log and will inform recruitment targets. No further information will be collected on ineligible patients or those that have not given consent for participation in the study.

Patient Recruitment

The PIS, developed with feedback from our Patient and Public Involvement (PPI) representatives, will be sent to the patient before the initial consultation, allowing time for the patient to review and ask questions. As part of the normal consultation process, the HCP will deliver a brief intervention detailing the importance of smoking cessation as per standard clinical practise (National Centre for Smoking Cessation Training [NCSCT] - Ask, Advice, Act). Patients will then be asked to consider and consent to a trial testing the new bespoke integrated smoking cessation support delivered in secondary care by the surgical/nursing team. If a patient does not wish to enter the INT arm they will be approached to take part in the UC arm of the study.

If patients want to join the trial on the day of the clinic appointment, they can do so, as the intervention is low risk and aimed at being offered 'there and then'. A research team member

For peer review only - http://bmjopen.bmj.com/site/about/guidelines.xhtml

will obtain written informed consent with delegated authority from the Principal Investigator. A copy of the signed consent form will be given to the participants and a copy will be placed in the medical notes. The original consent form will be stored in the investigators site file. Consent will be sought at every study contact and participants will be made aware that they are free to withdraw consent at any time without reprisal. Participants will be consented to inform their GP of their involvement in the study.

Intervention development

A smoking cessation package has been developed using best practice from NICE/NCSCT involving pharmacotherapy and a hybrid of behavioural support delivered by trained HCPs in the thoracic surgical pathway and a complementary web app. *'Quit4Surgery'* is a web app created using a user centred design approach and developed through a series of design workshops with patients, HCPs, academic researchers with expertise in smoking cessation and informed by behavioural frameworks/motivation theory. The iterative development was handled with 'Agile' and SCRUM project management (22, 23). *'Quit4Surgery'* contains behaviour change techniques that research suggests can improve the chances of quitting, including goal setting, self-monitoring, feedback, rewards, information about health consequences, advice on medication use, advice on changing routines, advice on coping and support for identity change (24). *'Quit4Surgery'* also enables HCPs to record and enter patients' CO levels.

Integration of full package of support into the surgical pathway (INT)

1) INT will be delivered by key HCPs in the surgical pathway (i.e. surgical nurses, lung cancer nurses, or pre-operative assessment nurses) who have received the NCSCT training to deliver behavioural support required for stop smoking practitioners with specific focus on major surgical patients (25). The initial consultation of 15-30 minutes will occur either on the day of consent or at the earliest convenience for the patient. This will be in a private room and will outline the benefits of stopping smoking before thoracic surgery, discuss pharmacotherapy options, provide behavioural support (26-29), decide on an early quit date (aim within 48 hours of consent) and provide further information regarding *'Quit4Surgery'*. The HCP will validate and quantify smoking amount using CO measurements, which will be repeated at subsequent face-to-face interactions, providing biofeedback upon the success of smoking cessation to the patient.

For peer review only - http://bmjopen.bmj.com/site/about/guidelines.xhtml

2) Pharmacotherapy is encouraged for all patients and includes either combined short- and long-acting nicotine replacement therapy (NRT) or varenicline, which are provided as standard care within the NHS. Patients wishing to use e-cigarettes will be given advice as per the NCSCT guidance (30). Treatment will be maintained for the peri-operative period to offset nicotine withdrawal symptoms and is typically 8-12 weeks for NRT and 12 weeks for varenicline as per NCSCT guidance, with pharmacotherapy and behavioural support tailored appropriately to individual patients.

3) *'Quit4Surgery'* will collect feedback regarding the patient's engagement, cravings and abstinence and provide motivational feedback to support the patient. The motivational feedback provided by the web app is guided by established behavioural change theory and will complement the patients smoking cessation package. Patient feedback using the web app to the surgical team will guide them as to the need for additional contact if the patient wishes. This will help improve overall efficacy of the intervention.

INT patients will receive proactive support within 2 days of the quit date (in person or by telephone) and then weekly until one month after surgery. The weekly sessions will concentrate on facilitators and barriers to quitting, relapse, pharmacotherapy side effects and withdrawal symptoms. Face-to-face interactions will occur during the surgical outpatient appointments, pre-clerking clinic and during hospital admission to reduce the number of additional visits. If patients are unable to attend face-to-face visits, videoconferencing or telephone visits will be attempted. Thus the INT will fit into the 'referral to treatment' target time frame and ensuring surgery is not delayed as recommended by NICE.

Control group of usual care (UC)

As part of the feasibility study, for those patients who decline consent to receive the intervention, we ask if they would consent to be observed during the thoracic surgical pathway. Termed usual care (UC), per usual local practice, patients are typically given a leaflet about the benefits of smoking cessation and referred to their local NHS smoking cessation service, which typically last up to 12 weeks and include pharmacotherapy. Patients will also be invited for an optional telephone interview after discharge. This is to help understand smoking cessation rates in usual care as well as the reasoning for non-participation in the intervention. Both groups will receive the same pre-, peri- and post-operative care as per protocol.

For peer review only - http://bmjopen.bmj.com/site/about/guidelines.xhtml

Withdrawal from the Trial

Withdrawal from the trial before surgery is a decision of the participant. However participants will be asked if the research team can still use the data collected during their participation in the research analyses.

Protocol Deviations

All study and protocol deviations will be documented in the patients CRF and reported to the Principle Investigator, who will notify the Sponsor of any serious breaches. Patients will be analysed according to group allocation, by intent-to-treat analysis.

Patient and Public Involvement

The project details were discussed at a national thoracic surgery patient group (RESOLVE) meeting and feedback regarding merit and acceptability of the proposed intervention were incorporated. Dissemination of results will occur via specific patient feedback events. A patient representative was an active contributor to the development of the trial and intervention and will be a member of the trial management group. The intervention including the web app *'Quit4Surgery'* were designed to meet patients' specific 'needs' and 'drivers' of smokers who are waiting for thoracic surgery, with a PPI group involved in the design and initial product testing stages of this feasibility study.

OUTCOME AND DATA COLLECTION

Patient Recruitment into study

The overall aims of the feasibility are to find out if a larger trial is feasible. The quantitative measurements related to this include:

- Proportion of all elective thoracic procedures screened
- Proportion of eligible participants of those screened
- Proportion of eligible participants consented to receive intervention

In this feasibility study, selecting 5 units whose overall elective thoracic patient throughput amounts to 4000 patients a year, of whom 20% are smokers and 20% agree to take part in

For peer review only - http://bmjopen.bmj.com/site/about/guidelines.xhtml

the study. It is expected that 60 eligible patients will be recruited to the INT group and 60 patients to the UC group of the study, with recruitment of 120 patients in total. Therefore the aim is to recruit 5 patients a month to each group over the 12 months recruitment period across all sites.

Patient Identification and Screening

The proportion of patients screened for eligibility and recorded on a screening log will be assessed and reported as the proportion of patients screened from the total number of planned major thoracic surgery during the study period.

Reasons for Failure to be Recruited

The proportion of patients that were missed, which should be minimal and proportion of patients who decline to take part will be recorded. Patients decline participation for many reasons, which should be captured whenever possible.

Education Material of Nurses and Surgeons

Feedback on the appropriateness, value and acceptability of the training will be elicited to enable refinement of the training programme for the substantive study, and to define a minimum competence. The training material will be evaluated for its ease of use, should it be used in the substantive study.

Assessment of Data Collection Process

Data will be collected using a CRF and will include demographic information and co-morbidities. Postoperative complications will be defined by the European Society of Thoracic Surgery (ESTS) (31), Thoracic Morbidity and Mortality (TM&M) system (32) and the stEP-COMPAC Group (33); see online supplementary appendix for definitions. Hospital re-admission rate will be determined within 30 days of discharge.

Assessment and identification will be made for loss of data during in-hospital stay to improve the data collection process for the substantive trial.

For peer review only - http://bmjopen.bmj.com/site/about/guidelines.xhtml
For peer review only

Smoking-related Outcomes

In the study the feasibility of the following questionnaires will be tested:

- Self-reported quit rate and exhaled CO measurement
- Fagerstrom Test for Nicotine Dependency: assessment of nicotine addiction (34).
- Mood and Physical Symptoms Scale (MPSS): assessment of cigarette withdrawal symptoms over the past 24 hours, including the strength of urge to smoke (35).
- Generic health related quality of life (EQ-5D-5L): assessment to provide a preference-based measure of health-related quality-of-life (36).
- NRT and support usage questionnaire.
- Health resource usage questionnaire: assessment of resource will be assessed via patient-recall with resources being measured including planned hospital overnight stays, planned hospital out-patient visits, hospital emergency visits, hospital admissions, GP and other community service visits.

Acceptability to and Impact on Patients

All patients consenting to participate in the trial will be eligible for interview and selected using maximum variety sampling by age, sex, ethnicity, admitting diagnosis, surgical procedure and smoking status. Interviews will be conducted until saturation is achieved, which is likely to be around 25-30 patients across all sites (37). Telephone interviews will be conducted as to minimise impact to patients following major surgery, will last no longer than 60 minutes and will be audio recorded and transcribed by the researcher.

An interview guide will be developed using evidence from previous experience of running the rehabilitation and pain study, and based on the interview objectives: pre- and post-surgical experiences of patients receiving the intervention (including effectiveness of staff communication) and patient engagement with it, any unintended consequences, acceptability of the intervention to patients, and recommendations for how its use or content/design could be improved.

Assessment of Trial Processes and Impact on Staff

Key HCPs in the clinical pathway will be invited to attend a focus group or individual interviews that will explore both the acceptability and recommendations for optimisation of the intervention.

For peer review only - http://bmjopen.bmj.com/site/about/guidelines.xhtml

Digitally recorded interviews and focus groups will be transcribed verbatim and anonymised. Transcripts will be analysed for patients and staff separately following Braun and Clarke's method for thematic analysis. Analysis will take an iterative approach, where data collection and analysis occurs concurrently, allowing the topic guide to be modified throughout to reflect emergent and/or priority themes.

STATISTICS AND DATA COLLECTION

Sample Size Calculation

As this is a feasibility study no formal sample size calculation has been performed. An audit discussing sample size in pilot and feasibility studies concluded that while sample size justification is important, formal calculation may not be appropriate. The findings from the audit concluded that a median size of 30 in each arm is appropriate (38). The study will aim to enrol 60 participants in the INT group over 1 year as this is a sufficient number to estimate a proportion of patients who have quit smoking by the day of surgery (39), as well as to explore data collection processes, and inform sample size calculations for a potential larger trial. Recruitment of 60 participants in each group will allow us to measure recruitment and compliance rates with 95% confidence interval (CI) width between 10 and 20%. It would also be enough to estimate the standard deviation (SD) of questionnaires with reasonable accuracy for future planning of a larger trial.

Data Analysis

Appropriate summary statistics (e.g. proportions and inter-quartile ranges, means and SDs) will be generated for the study feasibility and patient/clinical measures. Between-group measures (mean differences, etc.) will be generated alongside 95% CI, however formal hypothesis testing will not be carried out as the aim here is not to conclusively prove efficacy and furthermore the size of sample is too small for any inferential tests to be meaningful. Participants will be kept in the groups they were allocated, regardless of compliance with treatment (intention-to-treat protecting against attrition bias). Analysis will be completed once all participants have completed all follow-up assessments. All data used in publication will be in an anonymous format in order to maintain patient study participation confidential.

For peer review only

For peer review only - http://bmjopen.bmj.com/site/about/guidelines.xhtml

Handling Missing Data

A member of the research team will contact patients for any missing data (e.g. questionnaires) via telephone and post. Where patients attend for follow-up clinic, the potential for missing data will again be limited. Imputation of missing responses is not proposed for patient reported outcomes as this is not a definitive trial and no hypothesis testing will be performed.

DATA MANAGEMENT AND QUALITY ASSURANCE

Data management and confidentiality

Personal data will be collected from trial participants and hospital notes on CRFs, coded with the participant's unique trial number and initials. This will be held securely and strictly confidentially according to NHS policies. Patients in the semi-structured qualitative interviews will be consented specifically for their name, address and contact telephone number to be shared with University of Birmingham (UoB) and University College London. Data will be transferred securely by encrypted end-to-end email and will not be labelled with private identifiable information. Interview response information will be kept encrypted on a computer in a locked office. No data that could be used to identify an individual will be published. Data will be stored on a secure server under the provisions of the Data Protection Act and/or applicable laws and regulations. Data may be accessed by external regulatory agencies and the Study Sponsor representatives and permission for this access will be documented within the participants consent form.

Monitoring and Audit

Onsite monitoring will be conducted as required by the UoB Clinical Research Compliance Team, with activities reported to the trials team and any issues noted followed-up to resolution. Additional on-site monitoring visits may be triggered, e.g. by poor CRF return, poor data quality, low AE reporting rates, excessive number of participant withdrawals/deviations. Study data and evidence of monitoring and systems audits will be made available for inspection by the regulatory authority as required.

For peer review only - http://bmjopen.bmj.com/site/about/guidelines.xhtml

Long Term Storage of Data

Trial data will be stored archived after the formal closure of the trial in accordance with archive policy and for the appropriate duration as per current legislation.

Data access

Upon completion and publication of the study, individual participant data will be shared that underlie the results reported in study after de-identification. Additional related documents will be available including the study protocol, statistical analysis plan an analytical code. This data will be available in the beginning 3 months and 5 years following article publication to those who provide a methodologically sound proposal for analysis to achieve the aims in the approved proposal. All proposals should be directed to b.naidu@bham.ac.uk. To gain access requestors will need to sign a data access agreement. Data will be available for 5 years at a third party website.

SPONSORSHIP AND INDEMNITY

The UoB will act as the sponsor for this study. Delegated responsibilities will be assigned to the Chief Investigator and the NHS Trusts involved in the study. The UoB has in force a Public Liability Policy and/or Clinical Trials policy, which provides cover for claims for ‘negligent harm’ and the activities here are included within that coverage.

ETHICS AND DISSEMINATION

This study has obtained ethical approval from the NHS West Midlands Black Country Research Ethics Committee (Protocol Version 3.0; REC number 19/WM/0097). This aim of this feasibility study is to inform a substantive trial. On completion, results will be published in a peer-review scientific journal. The feasibility study is registered on the clinical trial database (NCT04190966).

For peer review only

REFERENCES

1. SCTS. National Thoracic Surgery Activity and Outcomes Report. 2018. https://scts.org/wp-content/uploads/2019/03/Thoracic-Blue-Book-2018-FINAL.pdf (accessed June 2020).
2. Agostini P, Lugg ST, Adams K, Vartsaba N, Kalkat MS, Rajesh PB, et al. Postoperative pulmonary complications and rehabilitation requirements following lobectomy: a propensity score matched study of patients undergoing video-assisted thoracoscopic surgery versus thoracotomydagger. *Interac Cardiovasc Thoracic Surg*. 2017;24(6):931-7.
3. Agostini P, Cieslik H, Rathinam S, Bishay E, Kalkat MS, Rajesh PB, et al. Postoperative pulmonary complications following thoracic surgery: are there any modifiable risk factors? *Thorax*. 2010;65(9):815-8.
4. Lugg ST, Tikka T, Agostini PJ, Kerr A, Adams K, Kalkat MS, et al. Smoking and timing of cessation on postoperative pulmonary complications after curative-intent lung cancer surgery. *J Cardiothorac Surg*. 2017;12(1):52.
5. Parsons A, Daley A, Begh R, Aveyard P. Influence of smoking cessation after diagnosis of early stage lung cancer on prognosis: systematic review of observational studies with meta-analysis. *BMJ*. 2010;340:b5569.
6. NICE. Smoking: acute acute maternity and mental health services. Public Health Guideline [PH48]: 2013. https://www.nice.org.uk/guidance/ph48 (accessed June 2020).
7. Thomsen T, Villebro N, Moller AM. Interventions for preoperative smoking cessation. *Cochrane Database Syst Rev*. 2014(3):CD002294.
8. Moller AM, Villebro N, Pedersen T, Tonnesen H. Effect of preoperative smoking intervention on postoperative complications: a randomised clinical trial. *Lancet*. 2002;359(9301):114-7.
9. Lindstrom D, Sadr Azodi O, Wladis A, Tonnesen H, Linder S, Nasell H, et al. Effects of a perioperative smoking cessation intervention on postoperative complications: a randomized trial. *Ann Surg*. 2008;248(5):739-45.
10. Webb J KA, Thickett D, Naidu B. Smoking habits of pre-surgery patients. *ERJ*. 2015;46: PA2511.
11. Warner DO, Klesges RC, Dale LC, Offord KP, Schroeder DR, Vickers KS, et al. Telephone quitlines to help surgical patients quit smoking patient and provider attitudes. *Am J Prev Med*. 2008;35(6 Suppl):S486-93.
12. Lee SM, Landry J, Jones PM, Buhrmann O, Morley-Forster P. Long-term quit rates after a perioperative smoking cessation randomized controlled trial. *Anesth and Analg*. 2015;120(3):582-7.
13. Farley A, Aveyard P, Kerr A, Naidu B, Dowswell G. Surgical lung cancer patients' views about smoking and support to quit after diagnosis: a qualitative study. *J Cancer Surviv*. 2016;10(2):312-9.
14. Park ER, Japuntich S, Temel J, Lanuti M, Pandiscio J, Hilgenberg J, et al. A smoking cessation intervention for thoracic surgery and oncology clinics: a pilot trial. *J Thoracic Oncol*. 2011;6(6):1059-65.
15. Rice VH, Heath L, Livingstone- Banks J, Hartmann- Boyce J. Nursing interventions for smoking cessation. *Cochrane Database Syst Rev*. 2017; Issue 12. Art. No: CD001188.
16. NICE. Lung cancer: diagnosis and management. NICE Guideline [CG121] 2011. https://www.nice.org.uk/guidance/CG121 (accessed June 2020).
17. National Cancer Waiting Times Monitoring Dataset Guidance – Version 9.0. 2015. http://www.swscn.org.uk/wp/wp-content/uploads/2018/11/Cancer-Waiting-Times-Guidance-v9.pdf (accessed June 2020)..
18. Thornton L, Quinn C, Birrell L, Guillaumier A, Shaw B, Forbes E, et al. Free smoking cessation mobile apps available in Australia: a quality review and content analysis. *Aust N Z J Public Health*. 2017;41(6):625-30.
19. McBride CM, Ostroff JS. Teachable moments for promoting smoking cessation: the context of cancer care and survivorship. *Cancer Control*. 2003;10(4):325-33.
20. Shi Y, Warner DO. Surgery as a teachable moment for smoking cessation. *Anesthesiology*. 2010;112(1):102-7.
21. Hemming K, Haines TP, Chilton PJ, Girling AJ, Lilford RJ. The stepped wedge cluster randomised trial: rationale, design, analysis, and reporting. *BMJ*. 2015;350:h391.
22. What is Scrum? An Agile Framework for Completing Complex Projects - Scrum Alliance. Scrum Alliance. 2016.
23. A M. Strategy, Implementation, Organisation and People. Springer Verlag. 2015.

For peer review only

24. Michie S, Hyder N, Walia A, West R. Development of a taxonomy of behaviour change techniques used in individual behavioural support for smoking cessation. *Addict Behav*. 2011;36(4):315-9.
25. NCSCT. Standard Treatment Programme. 2012. http://www.ncsct.co.uk/usr/pub/NCSCT%20STP.pdf (accessed June 2020).
26. West R, Sohal T. "Catastrophic" pathways to smoking cessation: findings from national survey. *BMJ*. 2006;332(7539):458-60.
27. Ong KC, Cheong GN, Prabhakaran L, Earnest A. Predictors of success in smoking cessation among hospitalized patients. *Respirology*. 2005;10(1):63-9.
28. Sivarajan Froelicher ES, Miller NH, Christopherson DJ, Martin K, Parker KM, Amonetti M, et al. High rates of sustained smoking cessation in women hospitalized with cardiovascular disease: the Women's Initiative for Nonsmoking (WINS). *Circulation*. 2004;109(5):587-93.
29. BTS. Recommendations for hospital smoking cessation services for commisioners and health care professionals. 2012. https://www.brit-thoracic.org.uk/media/70157/bts-recommendations-for-smoking-cessation-services_.pdf (accessed June 2020).
30. NCSCT. Electronic Cigarettes - A briefing for stop smoking services. 2016. https://www.ncsct.co.uk/usr/pub/Electronic_cigarettes._A_briefing_for_stop_smoking_services.pdf (accessed June 2020).
31. ESTS. Database Annual Report: Silver Book 2015. (accessed June 2020). http://www.ests.org/_userfiles/pages/files/ESTS%20201Silver_Book_FULL_PEF.pdf].
32. Seely AJ, Ivanovic J, Threader J, Al-Hussaini A, Al-Shehab D, Ramsay T, et al. Systematic classification of morbidity and mortality after thoracic surgery. *Ann Thorac Surg*. 2010;90(3):936-42; discussion 42.
33. Abbott TEF, Fowler AJ, Pelosi P, Gama de Abreu M, Moller AM, Canet J, et al. A systematic review and consensus definitions for standardised end-points in perioperative medicine: pulmonary complications. *B J Anaesth*. 2018;120(5):1066-79.
34. Heatherton TF, Kozlowski LT, Frecker RC, Fagerstrom KO. The Fagerstrom Test for Nicotine Dependence: a revision of the Fagerstrom Tolerance Questionnaire. *Br J Addict*. 1991;86(9):1119-27.
35. West R, Hajek P. Evaluation of the mood and physical symptoms scale (MPSS) to assess cigarette withdrawal. *Psychopharmacology*. 2004;177(1-2):195-9.
36. EuroQol--a new facility for the measurement of health-related quality of life. *Health Policy*. 1990;16(3):199-208.
37. Morse JM. Analytic Strategies and Sample Size. *Qual Health Res*. 2015;25(10):1317-8.
38. Billingham SA, Whitehead AL, Julious SA. An audit of sample sizes for pilot and feasibility trials being undertaken in the United Kingdom registered in the United Kingdom Clinical Research Network database. *BMC Med Res Methodol*. 2013;13:104.
39. Browne RH. On the use of a pilot sample for sample size determination. *Stat Med*. 1995;14(17):1933-40.

Author Contributions: All authors have made substantial contributions to the conception or design of the work, or acquisition, analysis or interpretation of the data. STL has participated in the study design, intervention development and drafted the manuscript. SK, AK and AB have participated in the intervention development. AF, OP, JB, RW, DRT, and BN have participated in the study design, intervention development and have critically revised the manuscript. All authors have approved the final version.

Funding: The research costs of the trial are funded by an investigator-initiated study grant from Johnson and Johnson (Grant #IIS-ENG-2017-01).

Competing Interests: None declared.

Word Count: 4062

For peer review only - http://bmjopen.bmj.com/site/about/guidelines.xhtml

Figure 1: Trial Schema

Table 1: Summary of investigations and assessments

	**Participants**		**Time points**					
	**Intervention Group (INT)**	**Usual Care Group (UC)**	**Baseline clinic prior to surgery**	**Day of surgery**	**During hospital Stay**	**Day of hospital discharge**	**One month after surgery**	**Six months after enrolment**
Eligibility and written informed consent	X	X	X					
Demographic Data[1]	X	X	X					
Previous Medical History[2]	X	X	X					
Self-reported Quit rate	X	X	X	X		X	X	X
Exhaled CO measurement	X	X	X	X		X	X	X
NRT and support usage questionnaire	X	X	X	X		X	X	X
Fagerstrom Test for Nicotine Dependence	X	X	X				X	
Mood and Physical Symptoms Scale	X		X	X		X	X	
EQ-5D-5L	X		X				X	
Health resource usage questionnaire	X						X	
Surgery and anesthetic data[3]	X	X		X				
Postoperative complications[4,5]	X	X			X	X	X	
Hospital readmission[6]	X	X					X	
Semi structured qualitative interviews[7]	X	X					X	
Adverse events	*If applicable*							
Protocol deviations	*If applicable*							

1) Demographic data: gender, age, indication for surgery, height, weight, BMI, ASA grade, ECOG score, dyspnoea score, recent lung function.
2) Previous medical history: smoking history, alcohol intake per week, co-morbidities (COPD, Ischaemic Heart Disease, Congestive Cardiac Failure, Hypertension, diabetes (diet-controlled/ oral therapy/ insulin), renal failure, previous stroke, thyroid disease (hyperthyroid/ hypothyroid).
3) Operation performed (side of surgery, operation, surgical technique).
4) **Post-operative data and observations**: Routine blood results if done (full blood count, albumin, renal function, electrolytes, CRP)

For peer review only

Acute complications: According to ESTS (30) (Appendix A) and Thoracic Morbidity and Mortality Classification (31) (Appendix B), data also collected including admission and length of stay on the ward (0), step-down (1), the HDU (2) and ITU (3). Data will also be collected in patients requiring mini-tracheostomy or additional surgery.

Post-operative pulmonary complications: Using stEP-COMPAC Group definition of postoperative pulmonary complications (32) (Appendix C) defining atelectasis (detected on computer tomography/CXR), pneumonia (using US Centres for Disease Control criteria), acute respiratory distress syndrome (using Berlin Consensus), and pulmonary aspiration (clear clinical history and radiological evidence).

5) Discharge data: total hospital stay, home with flutter bag, histology data and mortality.
6) Follow-up: hospital readmission up to and including one month following surgery.
7) At 4-8 weeks post-surgery patients will also have semi-structured qualitative patient interviews will be undertaken at 4 weeks post discharge to investigate experience, engagement, acceptability, unintended consequences/benefits and how to optimise the intervention delivery.

For peer review only

For peer review only - http://bmjopen.bmj.com/site/about/guidelines.xhtml

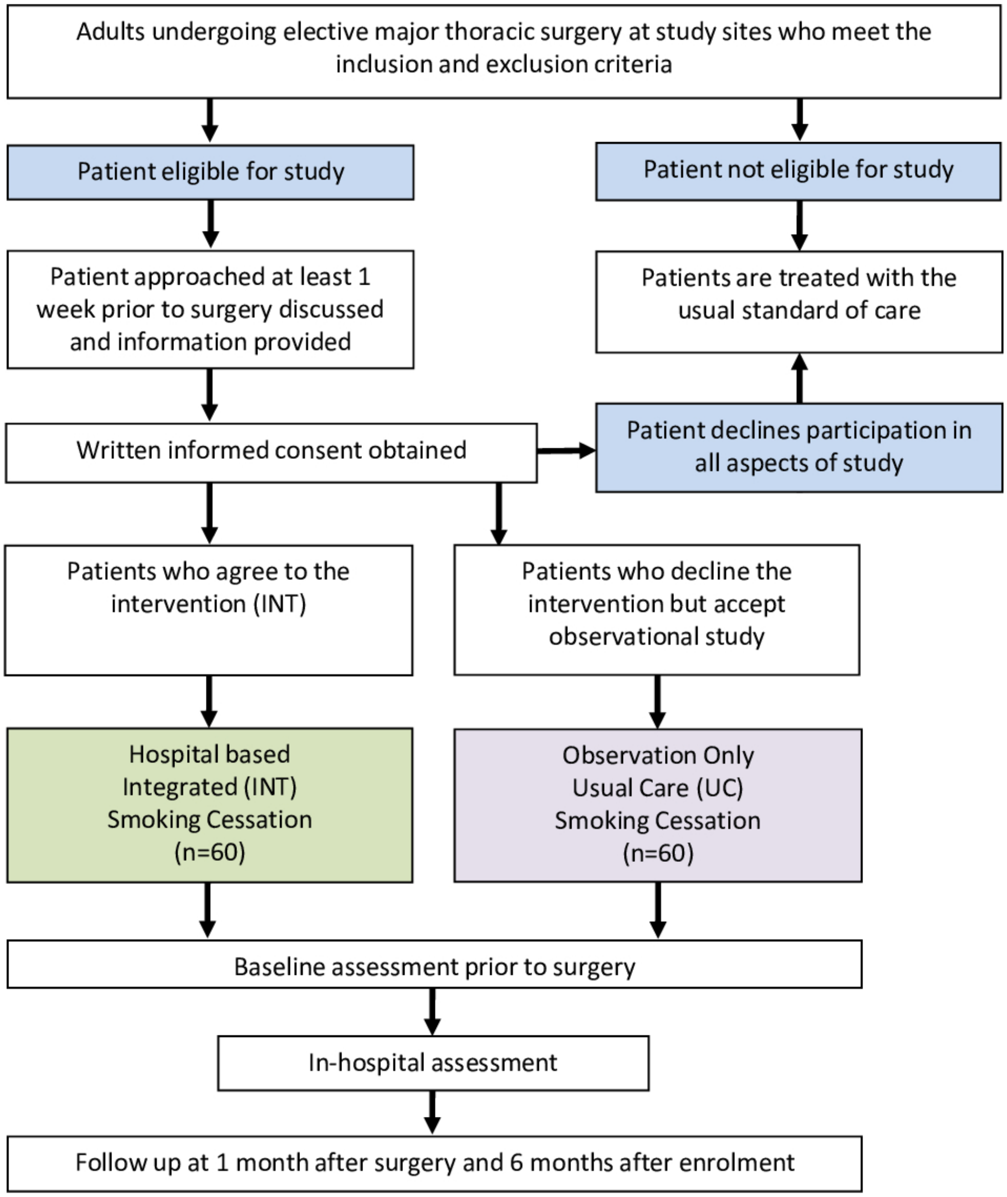

Trial Schema

80x95mm (300 x 300 DPI)

APPENDIX

Appendix A – Major cardiopulmonary complications as classified by the European Society of Thoracic Surgeons (ESTS)

ARDS: Adult respiratory distress syndrome defined according to the American-European consensus conference. All of the following criteria should be met:

1. Acute onset
2. Arterial hypoxemia with PaO2/FIO2 ratio lower than 200 (regardless PEEP level)
3. Bilateral infiltrates at chest radiograph or CT scan
4. No clinical evidence of left atrial hypertension or pulmonary artery occlusive pressure <18 mmHg
5. Compatible risk factors

Atrial Arrhythmia: new onset of atrial fibrillation/flutter (AF) requiring medical treatment or cardioversion. Does not include recurrence of AF which had been present preoperatively.

Ventricular Arrhythmia: sustained ventricular tachycardia or ventricular fibrillation that has been clinically documented and treated by ablation therapy, implantable cardioverter defibrillator, permanent pacemaker, pharmacologic treatment or cardioversion.

Bronchoscopy for atelectasis: postoperative atelectasis documented clinically or radiographically that needed bronchoscopy.

Pneumonia: defined according to the last CDC criteria. Two or more serial chest radiographs with at least **one** of the following:

- New or progressive and persistent infiltrate
- Consolidation
- Cavitation

AND at least **one** of the following:

- Fever (>38°C or >100.4°F) with no other recognized cause
- Leukopenia (<4000 WBC/mm3) or leukocytosis (>12,000 WBC/mm3)
- For adults >70 years old, altered mental status with no other recognized cause

AND at least **two** of the following:

- New onset of purulent sputum, or change in character of sputum, or increased respiratory secretions, or increased suctioning requirements
- New onset or worsening cough, or dyspnea, or tachypnea

For peer review only

- Rales or bronchial breath sounds Worsening gas exchange (e.g. O2 desaturations (e.g., PaO2/FiO2 < 240), increased oxygen requirements, or increased ventilator demand).

Pulmonary embolism: confirmed by V/Q scan, angiogram or CT scan.

DVT: deep venous thrombosis confirmed by Doppler study, contrast study or other study and that required treatment.

Myocardial infarct: evidenced by one of the following criteria:

1. Transmural infarction diagnosed by the appearance of a new Q wave in two or more contiguous leads on ECG.
2. Subendocardial infarction (non Q wave) evidenced by clinical, angiographic electrocardiographic signs.
3. Laboratory isoenzyme evidence of myocardial necrosis.

Renal failure: defined as the onset of new renal failure in the postoperative period according to one of the following criteria:

1. Increase of serum creatinine to greater than 2.0, and 2-fold the preoperative creatinine level.
2. A new requirement for dialysis postoperatively.

Neurological complication: occurrence of one of the following central neurologic postoperative events not present preoperatively:

1. A central neurologic deficit persisting postoperatively for more than 72 hours
2. A transient neurologic deficit (transient ischemic attack or reversible ischemic neurological deficit) with recovery within 72 hours
3. A new postoperative coma persisting at least 24 hours and caused by anoxic/ischemic and/or metabolic encephalopathy, thromboembolic event or cerebral bleed

For peer review only - http://bmjopen.bmj.com/site/about/guidelines.xhtml

Appendix B – Seeley Systematic Classification of Morbidity and Mortality After Thoracic Surgery (TM &M) Classification of Severity

Complication: Any deviation from the normal postoperative course.

**Minor**	
Grade I	Any complication without need for pharmacologic treatment or other intervention.
Grade II	Any complication that requires pharmacologic treatment or minor intervention only.
**Major**	
Grade III	Any complication that requires surgical, radiologic, endoscopic intervention, or multi-therapy.
Grade IIIa	Intervention does not require general anaesthesia.
Grade IIIb	Intervention requires general anaesthesia.
Grade IV	Any complication requiring intensive care unit management and life support.
Grade IVa	Single organ dysfunction.
Grade IVb	Multi-organ dysfunction.
**Mortality**	
Grade V	Any complication leading to the death of the patient.

For peer review only

Appendix C – StEP Core Outcome Measures in Perioperative and Anaesthetic Care (COMPAC) – Post-operative Pulmonary Complications

Post-operative Pulmonary Complications*

Composite of respiratory diagnoses that share common pathophysiological mechanisms including pulmonary collapse and airway contamination:

(i) atelectasis detected on computed tomography or chest radiograph,

(ii) pneumonia using US Centers for Disease Control criteria,

(iii) Acute Respiratory Distress Syndrome using Berlin consensus definition,

(iv) pulmonary aspiration (clear clinical history ***AND*** radiological evidence).

***Exclusions**

Other diagnoses that do not share a common biological mechanism are best evaluated separately and only when clearly relevant to the treatment under investigation:

(i) pulmonary embolism,

(ii) pleural effusion,

(iii) cardiogenic pulmonary oedema,

(iv) pneumothorax,

(v) bronchospasm.

ARDS - Berlin definition

Timing: within 1 week of a known clinical insult or new or worsening respiratory symptoms

AND Chest imaging: bilateral opacities not fully explained by effusions, lobar/lung collapse or nodules

AND Origin of oedema: respiratory failure not fully explained by cardiac failure or fluid overload (requires objective assessment, e.g. echocardiography, to exclude hydrostatic oedema),

AND Oxygenation:

Mild PaO_2:FiO_2 between 26.7 - 40.0 kPa (200-300 mm Hg) with PEEP or CPAP ≥ 5 cm H_2O;

Moderate PaO_2:FiO_2 between 13.3 - 26.6 kPa (100-200 mm Hg) with PEEP ≥ 5 cmH_2O;

Severe PaO_2:FiO_2 ≤ 13.3 kPa (100 mm Hg) with PEEP ≥ 5 cm H_2O.

Mechanical ventilation:

The need for need for tracheal re-intubation and mechanical ventilation after extubation, and within 30 days after surgery OR mechanical ventilation for more than 24 h after surgery. The inclusion of non-invasive ventilation may be considered on a study-by-study basis.

For peer review only - http://bmjopen.bmj.com/site/about/guidelines.xhtml

For peer review only

Post-operative Pneumonia

Two or more serial chest radiographs with at least one of the following (one radiograph is sufficient for patients with no underlying pulmonary or cardiac disease):
(i) New or progressive and persistent infiltrates,
(ii) consolidation
(iii) cavitation;

AND at least **one** of the following:
(a) fever (>38°C) with no other recognised cause,
(b) leucopaenia (white cell count <4x10^{9}/1) or leucocytosis (white cell count >12x10^{9}/l),
(c) for adults >70 years old, altered mental status with no other recognised cause;

AND at least **two** of the following:
(a) new onset of purulent sputum or change in character of sputum, or increased respiratory secretions, or increased suctioning requirements,
(b) new onset or worsening cough, or dyspnoea, or tachypnoea,
(c) rales or bronchial breath sounds,
(d) worsening gas exchange (hypoxaemia, increased oxygen requirement, increased ventilator demand).

For peer review only

For peer review only - http://bmjopen.bmj.com/site/about/guidelines.xhtml

[Insert Hospital Logo]

UNIVERSITY OF BIRMINGHAM

Study Number__________Site Number_________

A trial to study the effectiveness of sMoking cessation in the sURgical pathway befoRe mAjor lung sugerY: Project MURRAY: Feasibility Study

Participant Consent Form: Version 2.0

Principal Investigator:

Please initial box

1. I confirm that I have read and understand the patient information sheet (Version__Date__/__/__) for the above study. I have had the opportunity to consider the information, ask questions and have had these answered satisfactorily. ☐

2. I understand that my participation is voluntary and that I am free to withdraw at anytime without giving any reason, without my medical care or legal rights being affected. I give permission for data collected up until the point that I withdraw to be used in the research analysis. ☐

3. I understand that relevant sections of my medical notes and data collected during the study may be looked at by individuals from the research team, from regulatory authorities or from the NHS Trust, where it is relevant to my taking part in this research. I give permission for these individuals to have access to my records. ☐

4. I agree to be contacted either by telephone or post by a researcher to complete the research questionnaires at 4-8 weeks after surgery. ☐

5. I agree for my GP to be informed of my participation in the study. ☐

6. Optional – please choose option A) or B)

   A) I agree to take part in the interventional part of the study (including hospital based Intensive smoking cessation support). ☐

   B) I do not wish to take part in the interventional part of the study but I do agree to take part in the observational part of the study (including standard smoking cessation support). ☐

7. Optional - I agree for my name, address and contact details to be shared with the University of Birmingham and University College London, to be interviewed with audio recording and transcription at 4-8 weeks after surgery to discuss my treatment and participation in the study. I give permission that my anonymous quotes may be used in the reporting of the study. YES / NO ☐

8. I agree for my data to be used for ethically approved future research in lung surgery. ☐

_______________________ _____________ ___________________

Name of Participant **Date** **Signature**

_______________________ _____________ ___________________

Name of Person taking consent Date Signature

When completed: 1 for participant; 1 for researcher site file; 1 (original) to be kept in medical notes.

For peer review only - http://bmjopen.bmj.com/site/about/guidelines.xhtml

Insert Hospital Logo]

UNIVERSITY OF BIRMINGHAM

Study Number__________Site Number_________

A trial to study the effectiveness of sMoking cessation in the sURgical pathway befoRe mAjor lung sugerY Project MURRAY: Feasibility Study

Consent Form: Staff interview: Version 2.0

Principal Investigator:

Please initial box

1. I confirm that I have read and understand the participant information sheet Version ___ Date __/__/__ concerning my participation in an interview/focus group for the above study. I have had the opportunity to consider the information, ask questions and have had these answered satisfactorily. ☐

2. I understand that my participation is voluntary and that I am free to withdraw at anytime without giving any reason, without my medical care or legal rights being affected. I give permission for data collected up until the point that I withdraw to be used in the research analysis. ☐

3. I agree for my personal information including name, address and phone number to be shared with the University of Birmingham and University College London to be interviewed with audio recording and transcription. ☐

4. I give permission that my anonymous quotes may be used in the reporting of the study. ☐

5. I agree to be interviewed/participate in a focus group for the above study. ☐

____________________	____________________	____________________
**Name of Participant**	**Date**	**Signature**
____________________	____________________	____________________
Name of Person taking consent	Date	Signature

When completed: 1 for participant; 1 for researcher site file; 1 (original) to be kept in medical notes.

For peer review only - http://bmjopen.bmj.com/site/about/guidelines.xhtml

Reporting checklist for protocol of a clinical trial.

Based on the SPIRIT guidelines.

Instructions to authors

Complete this checklist by entering the page numbers from your manuscript where readers will find each of the items listed below.

Your article may not currently address all the items on the checklist. Please modify your text to include the missing information. If you are certain that an item does not apply, please write "n/a" and provide a short explanation.

Upload your completed checklist as an extra file when you submit to a journal.

In your methods section, say that you used the SPIRITreporting guidelines, and cite them as:

Chan A-W, Tetzlaff JM, Altman DG, Laupacis A, Gøtzsche PC, Krleža-Jerić K, Hróbjartsson A, Mann H, Dickersin K, Berlin J, Doré C, Parulekar W, Summerskill W, Groves T, Schulz K, Sox H, Rockhold FW, Rennie D, Moher D. SPIRIT 2013 Statement: Defining standard protocol items for clinical trials. Ann Intern Med. 2013;158(3):200-207

		Reporting Item	Page Number
**Administrative information**			
Title	#1	Descriptive title identifying the study design, population, interventions, and, if applicable, trial acronym	1
Trial registration	#2a	Trial identifier and registry name. If not yet registered, name of intended registry	1
Trial registration: data set	#2b	All items from the World Health Organization Trial Registration Data Set	1
Protocol version	#3	Date and version identifier	16
Funding	#4	Sources and types of financial, material, and other support	16
Roles and responsibilities: contributorship	#5a	Names, affiliations, and roles of protocol contributors	16

For peer review only

Roles and responsibilities: sponsor contact information	#5b	Name and contact information for the trial sponsor	1
Roles and responsibilities: sponsor and funder	#5c	Role of study sponsor and funders, if any, in study design; collection, management, analysis, and interpretation of data; writing of the report; and the decision to submit the report for publication, including whether they will have ultimate authority over any of these activities	16
Roles and responsibilities: committees	#5d	Composition, roles, and responsibilities of the coordinating centre, steering committee, endpoint adjudication committee, data management team, and other individuals or groups overseeing the trial, if applicable (see Item 21a for data monitoring committee)	16
**Introduction**			
Background and rationale	#6a	Description of research question and justification for undertaking the trial, including summary of relevant studies (published and unpublished) examining benefits and harms for each intervention	4
Background and rationale: choice of comparators	#6b	Explanation for choice of comparators	5
Objectives	#7	Specific objectives or hypotheses	6
Trial design	#8	Description of trial design including type of trial (eg, parallel group, crossover, factorial, single group), allocation ratio, and framework (eg, superiority, equivalence, non-inferiority, exploratory)	7
**Methods: Participants, interventions, and outcomes**			
Study setting	#9	Description of study settings (eg, community clinic, academic hospital) and list of countries where data will be collected. Reference to where list of study sites can be obtained	7

For peer review only

Eligibility criteria	#10	Inclusion and exclusion criteria for participants. If applicable, eligibility criteria for study centres and individuals who will perform the interventions (eg, surgeons, psychotherapists)	8
Interventions: description	#11a	Interventions for each group with sufficient detail to allow replication, including how and when they will be administered	9
Interventions: modifications	#11b	Criteria for discontinuing or modifying allocated interventions for a given trial participant (eg, drug dose change in response to harms, participant request, or improving / worsening disease)	9
Interventions: adherance	#11c	Strategies to improve adherence to intervention protocols, and any procedures for monitoring adherence (eg, drug tablet return; laboratory tests)	9
Interventions: concomitant care	#11d	Relevant concomitant care and interventions that are permitted or prohibited during the trial	9
Outcomes	#12	Primary, secondary, and other outcomes, including the specific measurement variable (eg, systolic blood pressure), analysis metric (eg, change from baseline, final value, time to event), method of aggregation (eg, median, proportion), and time point for each outcome. Explanation of the clinical relevance of chosen efficacy and harm outcomes is strongly recommended	6
Participant timeline	#13	Time schedule of enrolment, interventions (including any run-ins and washouts), assessments, and visits for participants. A schematic diagram is highly recommended (see Figure)	7
Sample size	#14	Estimated number of participants needed to achieve study objectives and how it was determined, including clinical and statistical assumptions supporting any sample size calculations	14
Recruitment	#15	Strategies for achieving adequate participant enrolment to reach target sample size	11
**Methods: Assignment of interventions (for controlled trials)**			
Allocation: sequence generation	#16a	Method of generating the allocation sequence (eg, computer-generated random numbers), and list of any factors for stratification. To reduce predictability of a random sequence, details of any planned restriction (eg, blocking) should be	n/a

For peer review only

		provided in a separate document that is unavailable to those who enrol participants or assign interventions	
Allocation concealment mechanism	#16b	Mechanism of implementing the allocation sequence (eg, central telephone; sequentially numbered, opaque, sealed envelopes), describing any steps to conceal the sequence until interventions are assigned	n/a
Allocation: implementation	#16c	Who will generate the allocation sequence, who will enrol participants, and who will assign participants to interventions	n/a
Blinding (masking)	#17a	Who will be blinded after assignment to interventions (eg, trial participants, care providers, outcome assessors, data analysts), and how	n/a
Blinding (masking): emergency unblinding	#17b	If blinded, circumstances under which unblinding is permissible, and procedure for revealing a participant's allocated intervention during the trial	n/a
**Methods: Data collection, management, and analysis**			
Data collection plan	#18a	Plans for assessment and collection of outcome, baseline, and other trial data, including any related processes to promote data quality (eg, duplicate measurements, training of assessors) and a description of study instruments (eg, questionnaires, laboratory tests) along with their reliability and validity, if known. Reference to where data collection forms can be found, if not in the protocol	12
Data collection plan: retention	#18b	Plans to promote participant retention and complete follow-up, including list of any outcome data to be collected for participants who discontinue or deviate from intervention protocols	14
Data management	#19	Plans for data entry, coding, security, and storage, including any related processes to promote data quality (eg, double data entry; range checks for data values). Reference to where details of data management procedures can be found, if not in the protocol	15
Statistics: outcomes	#20a	Statistical methods for analysing primary and secondary outcomes. Reference to where other details of the statistical analysis plan can be found, if not in the protocol	14

For peer review only

Statistics: additional analyses	#20b	Methods for any additional analyses (eg, subgroup and adjusted analyses)	14
Statistics: analysis population and missing data	#20c	Definition of analysis population relating to protocol non-adherence (eg, as randomised analysis), and any statistical methods to handle missing data (eg, multiple imputation)	14
**Methods: Monitoring**			
Data monitoring: formal committee	#21a	Composition of data monitoring committee (DMC); summary of its role and reporting structure; statement of whether it is independent from the sponsor and competing interests; and reference to where further details about its charter can be found, if not in the protocol. Alternatively, an explanation of why a DMC is not needed	15
Data monitoring: interim analysis	#21b	Description of any interim analyses and stopping guidelines, including who will have access to these interim results and make the final decision to terminate the trial	15
Harms	#22	Plans for collecting, assessing, reporting, and managing solicited and spontaneously reported adverse events and other unintended effects of trial interventions or trial conduct	15
Auditing	#23	Frequency and procedures for auditing trial conduct, if any, and whether the process will be independent from investigators and the sponsor	15
**Ethics and dissemination**			
Research ethics approval	#24	Plans for seeking research ethics committee / institutional review board (REC / IRB) approval	16
Protocol amendments	#25	Plans for communicating important protocol modifications (eg, changes to eligibility criteria, outcomes, analyses) to relevant parties (eg, investigators, REC / IRBs, trial participants, trial registries, journals, regulators)	16
Consent or assent	#26a	Who will obtain informed consent or assent from potential trial participants or authorised surrogates, and how (see Item 32)	15

For peer review only

Consent or assent: ancillary studies	#26b	Additional consent provisions for collection and use of participant data and biological specimens in ancillary studies, if applicable	15
Confidentiality	#27	How personal information about potential and enrolled participants will be collected, shared, and maintained in order to protect confidentiality before, during, and after the trial	15
Declaration of interests	#28	Financial and other competing interests for principal investigators for the overall trial and each study site	16
Data access	#29	Statement of who will have access to the final trial dataset, and disclosure of contractual agreements that limit such access for investigators	15
Ancillary and post trial care	#30	Provisions, if any, for ancillary and post-trial care, and for compensation to those who suffer harm from trial participation	16
Dissemination policy: trial results	#31a	Plans for investigators and sponsor to communicate trial results to participants, healthcare professionals, the public, and other relevant groups (eg, via publication, reporting in results databases, or other data sharing arrangements), including any publication restrictions	16
Dissemination policy: authorship	#31b	Authorship eligibility guidelines and any intended use of professional writers	16
Dissemination policy: reproducible research	#31c	Plans, if any, for granting public access to the full protocol, participant-level dataset, and statistical code	16
**Appendices**			
Informed consent materials	#32	Model consent form and other related documentation given to participants and authorised surrogates	Yes
Biological specimens	#33	Plans for collection, laboratory evaluation, and storage of biological specimens for genetic or molecular analysis in the current trial and for future use in ancillary studies, if applicable	n/a

The SPIRIT checklist is distributed under the terms of the Creative Commons Attribution License CC-BY-ND 3.0. This checklist was completed on 17. December 2019 using https://www.goodreports.org/, a tool made by the EQUATOR Network in collaboration with Penelope.ai

For peer review only
